# Therapeutic Effects of *Ganoderma lucidum* Against Aβ-Induced Neurotoxicity in SH-SY5Y Cells: In Vitro Evidence and Computational Validation of Human Protein Targets

**DOI:** 10.3390/molecules31183172

**Published:** 2026-09-09

**Authors:** Ece Miser-Salihoğlu, Mualla Pınar Elçi, Tuğba Fatsa, Sema Ören, Onur Kenan Ulutaş, Sevgi Yardim

**Affiliations:** 1 Department of Biochemistry, Faculty of Pharmacy, Gazi University, 06330 Ankara, Türkiye; sevgiy@gazi.edu.tr; 2 Institute of Stem Cell Lab, Faculty of Medicine, University of Health Sciences Gülhane, 06018 Ankara, Türkiye; muallapinar.elci@sbu.edu.tr; 3 Molecular Lab, Health Institute, University of Health Sciences Gülhane, 06018 Ankara, Türkiye; tugba.fatsa@sbu.edu.tr (T.F.); semaoren@gmail.com (S.Ö.); 4 Department of Toxicology, Faculty of Pharmacy, Gazi University, 06330 Ankara, Türkiye; onurkenan@gazi.edu.tr

**Keywords:** Alzheimer’s disease, molecular docking, Ganoderic Acid A, neuroinflammation, oxidative stress

## Abstract

The triterpenoid and polysaccharide constituents of *Ganoderma lucidum* (GL) are believed to influence key pathogenic pathways in Alzheimer’s disease (AD). This study first examined the molecular rationale for GL’s neuroprotective properties by subjecting its principal triterpenoids, Ganoderic Acid A&B, to computational profiling. Molecular docking was performed against human acetylcholinesterase (AChE), TNF-α, COX-2, IL-6, caspase-3, Bcl-2, and the Keap1–Nrf2 complex using CB-Dock2, alongside SwissADME-based physicochemical and ProTox-3.0-based toxicological screening. These targeted pathways were then biologically validated in an in vitro AD model induced by Aβ_1–42_ toxicity in SH-SY5Y cells, assessing AChE activity, apoptosis, ROS levels, mitochondrial membrane potential (MMP), and cytokine expression (COX-2, TGF-β1, IL-6, TNF-α, IL-10). Docking revealed high binding affinities of both triterpenoids toward all seven targets (Vina scores: −7.3 to −10.4 kcal/mol), predicting strong modulation of cholinergic, inflammatory, apoptotic, and antioxidant pathways. Consistent with these predictions, GL extract significantly reduced TNF-α, COX-2, and IL-6 mRNA and protein levels, attenuated ROS accumulation, preserved MMP except at 500 µg/mL, and exerted a concentration-dependent antiapoptotic effect. IL-10 and TGF-β1 showed complex, dose-dependent patterns, reflecting indirect regulatory responses. Together, these findings support GL’s neuroprotective potential against Aβ-induced toxicity through direct engagement of cholinergic, inflammatory, apoptotic, and antioxidant regulatory proteins.

## 1. Introduction

Alzheimer’s disease (AD) is a gradual, chronic neurological ailment marked by cognitive deterioration, impacting memory and logical reasoning [1]. The initial neurodegenerative damage generally manifests in brain regions associated with memory, language, and cognition; hence, the primary symptoms often involve deficits in these domains.

The global burden of Alzheimer’s disease and other dementias has increased over recent decades, with approximately 56.9 million prevalent cases and 9.8 million new cases reported worldwide in 2021 [2]. Presently, around 47 million individuals are diagnosed with dementia globally, with projections of 76 million by 2030 and 135.5 million by 2050 [3,4]. The socioeconomic burden continues to increase with the growing prevalence of AD. Although several therapeutic options are currently available, there is still no curative treatment for AD. Current pharmacological treatments may provide symptomatic benefits and, in some cases, slow disease progression [5].

The pathophysiology of Alzheimer’s disease is characterized primarily by the aggregation of β-amyloid (Aβ) peptides into amyloid plaques and the formation of neurofibrillary tangles (NFTs) resulting from the hyperphosphorylation of the Tau protein. Such accumulation can lead to oxidative stress, loss of membrane integrity, mitochondrial dysfunction, disruptions in the cell cycle, protein misfolding, and DNA damage [6]. The accumulation of these abnormal proteins contributes to neuronal degeneration and synaptic loss. Synaptic dysfunction is considered one of the earliest and most important pathological events in AD and is closely associated with neuronal loss, oxidative stress, and neuroinflammatory responses [7]. Synaptic toxicity elevates TNF-α levels and activates glutamate receptors. TNF-α and FAS ligands bind their respective receptors, FAS and TNF receptor I (TNFRI), initiating a cascade involving NF-κB and caspase-8. Caspase-8 also activates caspase-9 and effector caspase-3, leading to DNA damage and neuronal death. Overstimulation of the excitatory glutamate receptor opens Ca^2+^ channels, leading to increased Ca^2+^ influx, activation of nitric oxide synthase (NOS), and excessive production of reactive oxygen species (ROS) and nitric oxide (NO). The production of ROS leads to mitochondrial membrane damage, releasing cytochrome c. This induces APAF-1 activation, subsequently initiating a caspase cascade via caspase-9, leading to DNA damage and neuronal apoptosis [8]. Moreover, COX-2 activation promotes NF-κB transactivation and IL-1β production, and mediates bidirectional signaling between neuronal and glial cells that controls Aβ and IL-1β [9].

Three major interconnected processes involved in AD pathology are excitotoxicity, oxidative stress, and neuroinflammation. Excitotoxicity and oxidative stress induce neuroinflammation, which, in turn, perpetuates the inflammatory response through a negative feedback loop. It accomplishes this by prompting modifications in peripheral cytokine secretion, trauma, and stress [9,10]. Excitotoxicity predominantly results from excessive accumulation of glutamate in synaptic clefts, leading to prolonged receptor activation, calcium overload, mitochondrial dysfunction, and eventual neuronal death. These processes contribute to the development of excitotoxicity, oxidative stress, and neuroinflammation. Although synaptic impairment is central to AD pathology, many of its underlying mechanisms are driven by molecular and cellular processes, including oxidative stress, inflammatory cytokine release, mitochondrial dysfunction, and apoptosis. Therefore, in vitro neuronal models provide a valuable platform for investigating these indirect yet critical contributors to synaptic dysfunction under controlled experimental conditions.

*Ganoderma lucidum*, a Basidiomycete fungus in the family Polyporaceae, has been widely used in traditional Asian medicine for centuries for its longevity-promoting, cognitive-enhancing, and health-supporting properties. In addition to its ethnopharmacological importance, GL has gained considerable attention as a functional food and nutraceutical and is widely incorporated into dietary supplements, functional beverages, and health-promoting formulations. Its fruiting body, mycelium, and spores contain nearly 400 bioactive constituents, including polysaccharides, triterpenoids, sterols, nucleosides, peptides, phenolic compounds, fatty acids, alkaloids, polypeptides, and inorganic elements. Owing to this rich phytochemical composition, GL exhibits a broad spectrum of pharmacological activities, including antioxidant, anti-inflammatory, immunomodulatory, antibacterial, antiviral, anti-aging, and neuroprotective effects. Consequently, it has traditionally been referred to as the “Mushroom of Immortality,” the “Divine Herb,” the “Spiritual Power Plant,” and the “Heavenly Herb” [11,12,13,14]. Beyond its traditional reputation as a neurotonic and anti-aging agent, increasing experimental evidence suggests that the triterpenoid and polysaccharide constituents of GL may modulate key pathological processes involved in Alzheimer’s disease, including oxidative stress, neuroinflammation, β-amyloid accumulation, and neuronal apoptosis.

Although the anti-inflammatory, antioxidant, and antiapoptotic properties of GL have been extensively documented [13,14,15], comparatively less attention has been given to the potential interactions between its major bioactive triterpenoids and human protein targets involved in these pathways using structure-based computational approaches. Ganoderic Acid A and Ganoderenic Acid B, among the most abundant lanostane-type triterpenoids in GL fruiting body extracts, have been proposed as key mediators of its pharmacological activity [13,15]. Elucidating their binding behavior toward human acetylcholinesterase [16], pro-inflammatory cytokines TNF-α [17] and IL-6 [18], COX-2 [19], apoptosis-regulatory proteins caspase-3 [20] and Bcl-2 [21], and the Keap1–Nrf2 antioxidant complex [22] may therefore provide mechanistic evidence that directly corroborates the functional outcomes observed in cellular assays.

The primary objective of this study was to systematically elucidate the therapeutic mechanisms of a GL Fruiting Body Dry Extract USP Reference Standard extract by first identifying the direct molecular targets of its active constituents and subsequently functionally validating these specific pathways. Accordingly, this study initiates with in silico molecular docking, ADME, and toxicological profiling of GL’s major triterpenoids (Ganoderic Acid A and Ganoderenic Acid B) against key human proteins involved in cholinergic signaling, neuroinflammation, apoptosis, and oxidative stress. These computational findings were combined with experimental assessments of cell viability, apoptosis, oxidative stress, mitochondrial membrane potential, acetylcholinesterase (AChE) activity, and inflammatory mediators in an Aβ_1–42_-induced SH-SY5Y cell model. This comprehensive approach facilitates the integration of predicted molecular interactions with observed cellular responses, offering a broader perspective on the potential therapeutic effects of GL in AD.

## 2. Materials and Methods

### 2.1. Cell Culture

The cell line used in our study is SH-SY5Y (ATCC^®^ CRL-2266™), a human neuroblastoma cell line obtained from the American Type Culture Collection (ATCC, Manassas, VA, USA). DMEM containing 100 μg/mL penicillin, 10% FBS (Invitrogen, Burlington, ON, Canada), 1% L-glutamine, and 100 μg/mL streptomycin was used to cultivate human neuroblastoma SH-SY5Y cells at 37 °C with 5% CO_2_. Cell viability and count were evaluated using the EVE Cell Counter (NANOENTEK, Cat no. EVE-MC, Serial num. E14111-019, Seoul, Republic of Korea). Mycoplasma contamination was assessed using the MycoStrip^®^ Mycoplasma Detection Kit (InvivoGen, Cat.code: rep-mysv2-10, San Diego, CA, USA), and all cell cultures were confirmed to be mycoplasma-free prior to the experiments. SH-SY5Y cells were used in their undifferentiated state throughout all experiments, and no differentiation protocol was applied prior to Aβ_1–42_ or GL extract treatment.

### 2.2. Preparation of Aβ_1–42_

A 4 mM stock solution was prepared by dissolving 1 mg of Aβ_1–42_ (Item No. 20574, Cayman, Ann Arbor, MI, USA) in 55.4 mL of DMSO. The stock solution (diluted in DMEM medium) was administered at doses of 2.5, 5, 10, 50, 100, 200, and 400 µM and applied for 24, 48, and 72 h. IC50 values of Aβ_1–42_ were found to be in the range of 100 µM [23].

### 2.3. In Vitro Alzheimer’s Disease Model

In line with previous studies, a 100 μM Aβ_1–42_ dose was selected to induce the in vitro AD model [24,25,26,27,28]. Following the Aβ_1–42_ treatment, 100 μL of MTT (5 mg/mL) (SERVA, Lot: 190184, Heidelberg, Germany) solution was added, and the cells were incubated for 4 h. The supernatant was aspirated, and 100 μL of isopropyl alcohol was added. After dissolving the formazan crystals in 100 μL of isopropyl alcohol and incubating in the dark for two hours, absorbance at 570 nm was recorded. The IC50 (inhibition concentration at which cells show 50% viability) of Aβ_1–42_ was determined to be 100 μM. All procedures were carried out three times.

### 2.4. Preparation of GL Extract

The GL fruiting body dry extract (USP Reference Standard; Cat. No. 1288372, Lot F012B0, Darmstadt, Germany) used in this study is a commercially available standardized reference material. According to the official USP certificate, the extract contains multiple triterpenoid constituents, including Ganoderic Acid A and Ganoderenic Acid B, and is characterized in accordance with the identification and triterpenoic acid content procedures described in the USP Herbal Medicines Compendium. However, the individual quantitative contents of Ganoderic Acid A and Ganoderenic Acid B are not specified in the USP certificate. The USP reference standard certificate and the corresponding representative chromatographic data are provided in the Supplementary Materials.

The GL extract was prepared according to the pharmacopoeial procedure. Briefly, 1 g of GL was sonicated in 50 mL of 95% methanol for 15 min. After centrifugation, the supernatant was removed and evaporated to dryness at 50 °C under low pressure. The residue was reconstituted in 2.0 mL of methanol and centrifuged before experimental use [29].

### 2.5. MTT Assay for GL Extracts

Cells were pre-treated with 100 μM Aβ_1–42_ in 200 μL volumes for 24 h. After that, GL extract was administered at doses ranging from 31.25 to 1000 µg/mL and was applied for 24, 48, and 72 h. Following treatment, 100 μL of a 5 mg/mL MTT solution was added, and the supernatant was aspirated after 4 h of incubation. After dissolving the formazan crystals in 100 μL of isopropyl alcohol and incubating in the dark for two hours, absorbance at 570 nm was recorded. All procedures were carried out three times.

### 2.6. AChE Level

AChE levels were determined using an ELISA kit (Elabscience, Cat. No. E-EL-H6031, Lot No. AK18DNBNX6849, Houston, TX, USA) according to the manufacturer’s instructions. The wells were sequentially treated with biotinylated detection antibody, HRP conjugate, and substrate after a 90-min incubation at 37 °C with 100 µL of standard and sample. After adding the stop solution, the measurement was taken at 450 nm.

#### Experimental Groups

The control (C) group consisted of untreated cells, and the Aβ control (Aβ-C) consisted of cells exposed to 100 μM Aβ_1–42_ [21] for 24 h to establish an in vitro AD model. The experimental groups were first exposed to 100 μM Aβ_1–42_ for 24 h, then treated with GL extract at concentrations of 31.25, 62.5, 125, 250, and 500 μg/mL for an additional 24 h. These groups were used for all subsequent biochemical and molecular analyses.

The final concentration of DMSO in the culture medium was maintained below 0.1%. Methanol used during GL extract preparation was removed by evaporation before experimental use. Therefore, the observed effects were attributed to the GL extract rather than residual extraction solvent.

### 2.7. Apoptosis

Apoptosis was assessed in SH-SY5Y cells (5 × 10^5^ per group) harvested by trypsinization. After being resuspended in binding buffer, cells were treated with 5 μL Annexin V and 20 μL propidium iodide for 30 min at RT in the dark. After adding 400 μL of 1X binding buffer, cells were examined by BD Accuri™ C6 Plus flow cytometry (BD Bioscience, San Jose, CA, USA), and Annexin V+/PI− cells were considered apoptotic.

### 2.8. Mitochondrial Membrane Potential

As a measure of cell viability, MMP (ΔΨm) is a crucial component of mitochondrial activity. One of the earliest intracellular events occurring during apoptosis induction is the disruption of mitochondrial transmembrane potential. The Δψm was assessed by quantifying the fluorescence intensity of cells labeled with JC-1 dye. JC-1 staining solution (5 µg/mL) was used to incubate SH-SY5Y cells exposed to various concentrations of GL extract (500-250-125-62.5-31.25 µg/mL) for 45 min at 37 °C. The JC-1 staining buffer was used twice to wash the cells. After incubation, the cells were washed twice with JC-1 staining buffer. The fluorescence signal was quantified by spectrofluorometry, using excitation/emission settings of 514/529 nm for green (monomeric) fluorescence and 585/590 nm for red (aggregated) fluorescence. The ΔΨm of cells was quantified for each group by the red/green fluorescence ratio.

### 2.9. ROS Levels

The ROS Detection Kit (THORVACS Biotechnology, Cat# ROS-100T, Ankara, Türkiye) was used to determine ROS levels. Cells from the experimental groups were centrifuged at 300× *g* for five minutes. They were then dissolved in 1X PBS for washing and centrifuged again. The cell pellet was resuspended in cell medium with 1X dye solution (100 mM DCF-DA reagent) following the removal of the supernatant. The samples were examined using the FL1 channel on the BD Accuri C6 flow cytometer after verifying that the cells were fully dissociated and that there were no stuck cells in the media, and were incubated for 30 min.

### 2.10. mRNA Expressions of Cytokines

To evaluate the effect of mushroom extract on pro- and anti-inflammatory cytokines, the gene expression of TNF-α, COX-2, IL-6, IL-10, and TGF-β1 was studied. First, ribonucleic acid (RNA) was obtained using a ready-made mRNA isolation kit, and RNA purity and quantity were determined using a NanoDrop 2000 spectrophotometer (Thermo Fisher Scientific, Waltham, MA, USA) based on the OD260/OD280 nm ratio. The effects of the mushroom extract on the expression of pro- and anti-inflammatory cytokines (TNF-α, COX-2, IL-6 mRNA, IL-10, and TGF-β1 mRNA) were evaluated by RT-qPCR. GAPDH was used as the housekeeping gene in the study (Table 1). The PCR was performed for 40 cycles with the following conditions: denaturation at 95 °C for 15 s, annealing at 56 °C for 30 s, and extension at 72 °C for 30 s. The 2^−ΔΔCT^ was used to calculate the results.

### 2.11. Protein Levels of Cytokines

To evaluate the effect of mushroom extract on pro- and anti-inflammatory cytokines, TNF-α (Elabscience, Cat. No. E-EL-H0109, Lot No. WV52LN2L85628, Houston, TX, USA), COX-2 (BT LAB, Cat. No. E4613Hu, Lot No. 202502008, Jiaxing, Zhejiang, China), IL-6 (Elabscience, Cat. No. E-EL-H6154, Lot No. WV16D8RX5069, Houston, TX, USA), IL-10 (Elabscience, Cat. No. E-EL-H6154, Lot No. WV55L26ZD2869, Houston, TX, USA), and TGF-β1 (Elabscience, Cat. No. E-EL-H10110, Lot No. WW36L4BB033333, Houston, TX, USA), gene expression analysis was performed. Each parameter was measured using commercial kits in compliance with the manufacturer’s guidelines.

### 2.12. Molecular Docking Analysis

The three-dimensional structures of Ganoderic Acid A and Ganoderenic Acid B, the principal triterpenoid constituents of the GL extract used in this study, were docked against seven human protein targets selected to correspond directly with the in vitro endpoints assessed: acetylcholinesterase (AChE; PDB: 4EY7) [16], tumor necrosis factor-alpha (TNF-α; PDB: 2AZ5) [17], interleukin-6 (IL-6; PDB: 1ALU) [18], cyclooxygenase-2 (COX-2; PDB: 5F19) [19], caspase-3 (PDB: 2XYG) [20], B-cell lymphoma 2 protein (Bcl-2; PDB: 4AQ3) [21], and the Kelch domain of Keap1 in complex with Nrf2 (PDB: 4IN4) [22]. All selected structures were confirmed to be of human (Homo sapiens) origin via the RCSB Protein Data Bank. Blind docking was performed using the CB-Dock2 server, which employs automated cavity detection (CurPocket algorithm) followed by AutoDock Vina (v1.1.2/v1.2.0) based scoring [30]. For each target, the five top-ranked binding cavities were evaluated based on predicted cavity volume, docking box center coordinates, and Vina binding energy scores, and the cavity exhibiting the most favorable score was cross-checked against the reported catalytic or ligand-binding residues of each protein including the AChE catalytic gorge [16], the COX-2 Ser530 acetylation site [19], and the Keap1 Kelch domain DxETGE-binding pocket [22] to confirm the biological relevance of the identified pocket.

### 2.13. Physicochemical and Toxicological Profiling

Physicochemical descriptors, lipophilicity, water solubility, and pharmacokinetic parameters of Ganoderic Acid A and Ganoderenic Acid B were predicted using the SwissADME web tool [15]. Toxicological endpoints, including predicted median lethal dose (LD50) and toxicity class, were assessed using ProTox-3.0 [31].

### 2.14. Data Analysis

All experiments were repeated three times, and data are presented as mean ± SD. The suitability of the continuous data for a normal distribution was assessed using the Kolmogorov–Smirnov test. The distributions of variables that did not follow a normal distribution in three or more groups were analyzed using the Kruskal–Wallis test. When a significant difference was detected, pairwise comparisons were performed using Dunn’s post hoc test with Bonferroni correction. Data were analyzed using IBM SPSS Statistics 27 (IBM SPSS Inc., Chicago, IL, USA). A *p*-value < 0.05 was considered statistically significant.

## 3. Results

### 3.1. Molecular Docking, ADME, and Toxicity Profiling

Molecular docking analysis demonstrated that both Ganoderic Acid A and Ganoderenic Acid B exhibited favorable binding affinities toward all seven selected human protein targets (Table 2). The strongest interactions for both triterpenoids were observed with the Keap1 Kelch domain (PDB: 4IN4; −10.4 kcal/mol for Ganoderic Acid A, −10.1 kcal/mol for Ganoderenic Acid B) and human COX-2 (PDB: 5F19; −10.1 kcal/mol for Ganoderic Acid A, −10.2 kcal/mol for Ganoderenic Acid B). Binding to AChE (PDB: 4EY7) yielded Vina scores of −9.4 and −9.0 kcal/mol for Ganoderic Acid A and Ganoderenic Acid B, respectively, while interactions with Bcl-2 (PDB: 4AQ3) yielded −9.2 and −8.6 kcal/mol. Both triterpenoids showed comparable affinities toward caspase-3 (PDB: 2XYG; −8.5 and −7.8 kcal/mol), TNF-α (PDB: 2AZ5; −7.5 and −7.8 kcal/mol), and IL-6 (PDB: 1ALU; −7.3 kcal/mol for both compounds). SwissADME analysis indicated that both compounds exhibited low gastrointestinal absorption and lacked blood–brain barrier permeability, consistent with their moderate aqueous solubility profiles [15], while ProTox-3.0 predictions indicated a predicted rodent oral LD50 of 9000 mg/kg and a Toxicity Class of 6 for both triterpenoids, corresponding to a practically non-toxic classification [30]. Both compounds were predicted to be inactive for hepatotoxicity, neurotoxicity, and mutagenicity, whereas nephrotoxicity, respiratory toxicity, cardiotoxicity, and carcinogenicity endpoints were predicted as active with low-to-moderate probabilities (0.50–0.96). Notably, Ganoderenic Acid B, but not Ganoderic Acid A, was predicted to exhibit immunotoxic potential (probability 0.83 versus 0.52, respectively), a compound-specific distinction warranting further experimental verification.

### 3.2. AD Model

**Figure 1:**
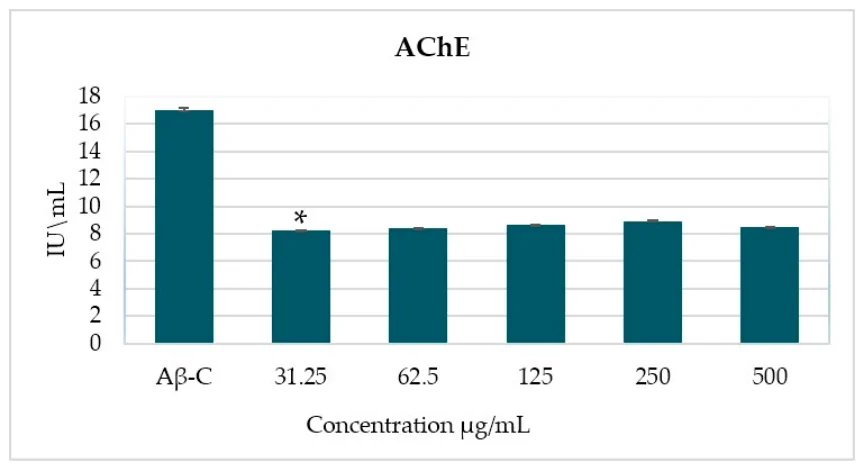
AChE protein levels in SH-SY5Y cells following Aβ_1–42_ exposure and subsequent treatment with GL extract. * *p* < 0.01 compared with the Aβ Control group.

Prompted by the favorable in silico binding profiles of Ganoderic Acid A (−9.4 kcal/mol) and Ganoderenic Acid B (−9.0 kcal/mol) with human AChE, AChE protein levels were subsequently evaluated in vitro. Following exposure of SH-SY5Y cells to 100 μM Aβ_1–42_, AChE protein levels were measured using ELISA to assess the effect of Aβ_1–42_ exposure and the subsequent response to GL treatment. Aβ_1–42_ increased AChE protein levels by 62.47% in the Aβ control (Aβ-C) group relative to the control (C) group, supporting the establishment of the Aβ_1–42_-induced cellular model. GL treatment reduced AChE protein levels compared with the Aβ-C group across all tested concentrations. The predicted binding of Ganoderic Acid A and Ganoderenic Acid B to human AChE (PDB: 4EY7) [16] provides complementary computational evidence for a potential interaction with this target. The Kruskal–Wallis test revealed a significant difference among the groups (χ^2^ = 19.636, *p* < 0.005). A statistically significant reduction in AChE protein levels was observed at 31.25 µg/mL compared with the Aβ-C group (*p* < 0.01) (Figure 1).

### 3.3. MTT Cell Viability Test

**Figure 2:**
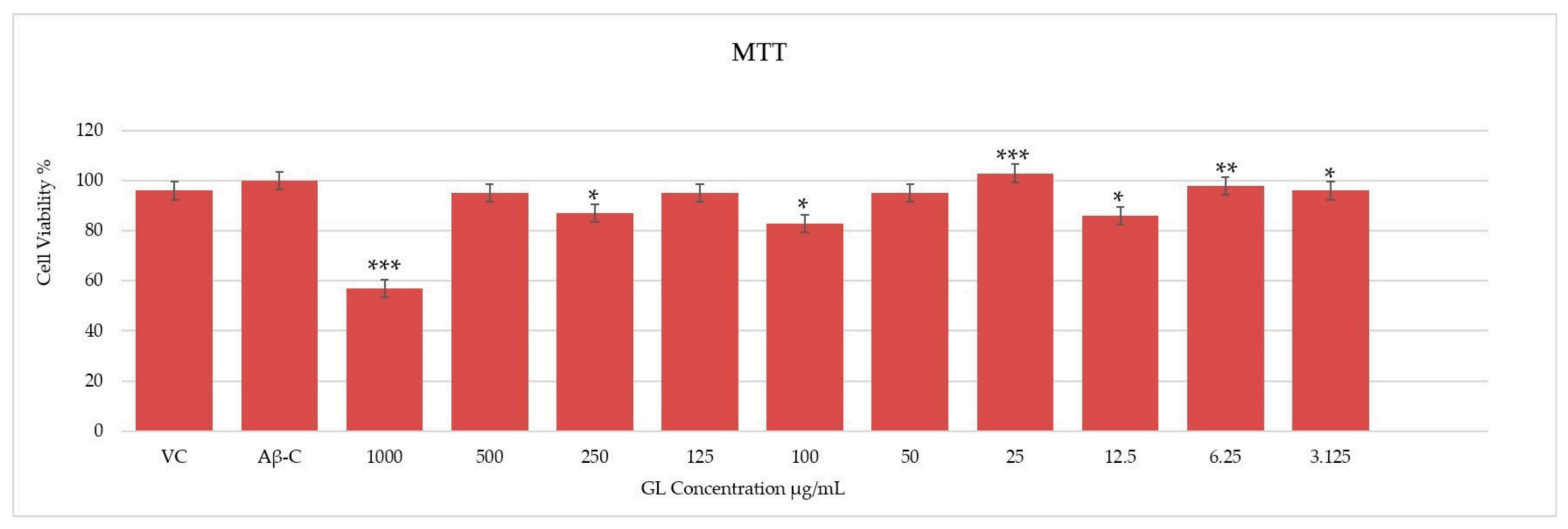
Effect of GL treatment on cell viability in Aβ_1–42_-induced SH-SY5Y cells after 24 h. Cell viability was assessed using the MTT assay. Data are presented as mean ± SD. Statistical differences among groups were evaluated using the Kruskal–Wallis test followed by pairwise comparisons with Bonferroni correction. * *p* < 0.05, ** *p* < 0.01, and *** *p* < 0.001 compared with the Aβ-C group.

GL extract showed concentration- and time-related changes in the viability of Aβ_1–42_-treated SH-SY5Y cells at concentrations ranging from 3.125 to 1000 µg/mL after 24, 48, and 72 h (Figure 2).

Cell viability at 1 mg/mL was lower than at the other concentrations. At 48 and 72 h, excessive cell proliferation was observed, reducing the inhibitory effect of Aβ_1–42_ on cell proliferation. Therefore, 24-h application and concentrations of 500, 250, 125, 62.5, and 31.25 µg/mL were selected. The selected doses and 24-h application were applied to the in vitro AD model created in SH-SY5Y cells with Aβ_1-42_-at 24, 48, and 72 h, the means of the GL-treated groups were compared with the control group without GL, showing statistically significant differences (F(10,55) = 7.125, *p* < 0.000; F(10,55) = 3.828, *p* < 0.001; F(10,55) = 6.362, *p* < 0.000, respectively). Following multiple comparison testing, the 1 mg/mL group exhibited a significantly lower mean compared to the other groups (*p* < 0.001) (Figure 2).

### 3.4. Annexin-V Analysis

**Figure 3:**
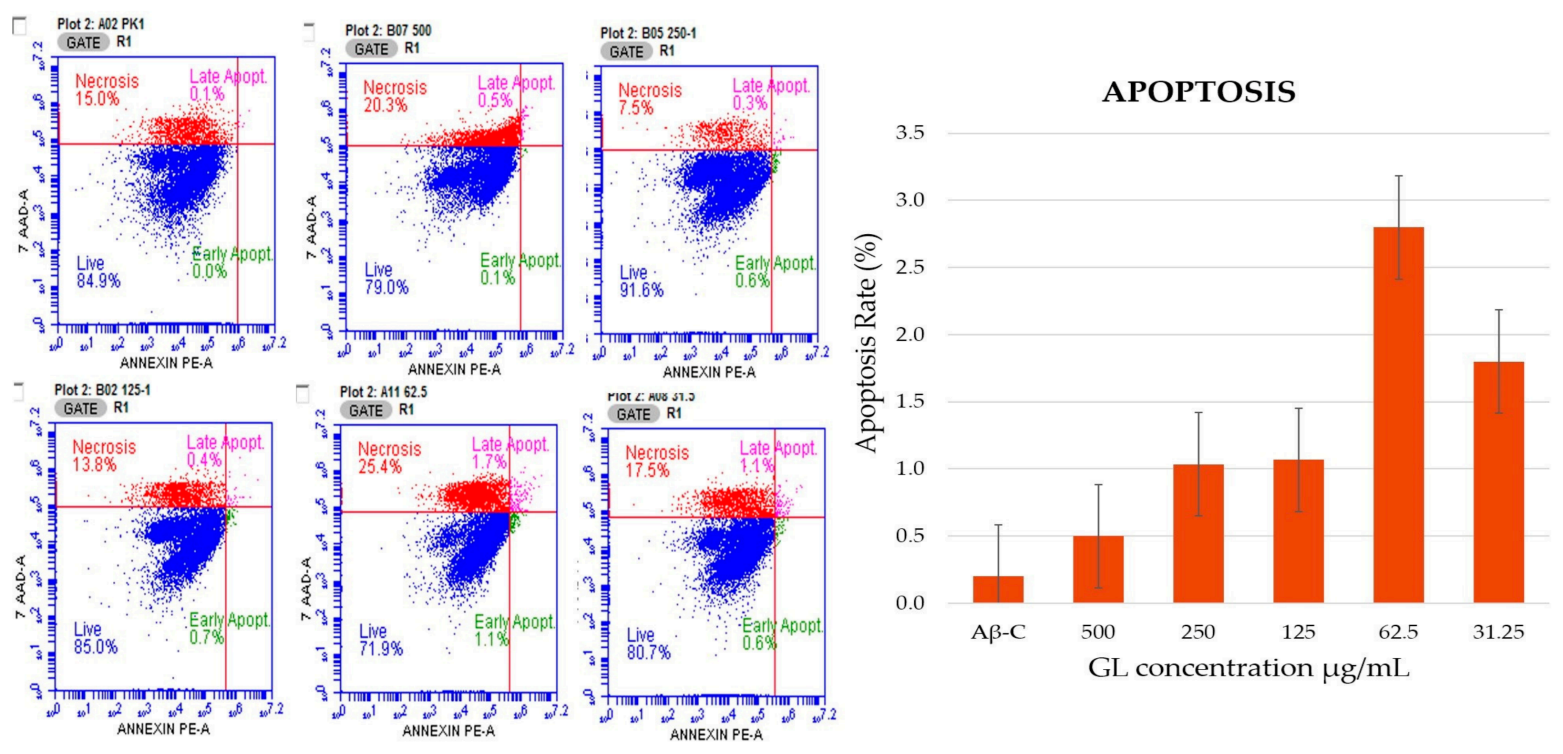
Annexin V/PI flow cytometric analysis of apoptosis in Aβ_1–42_-treated SH-SY5Y cells following treatment with GL extract (31.25–500 µg/mL) for 24 h. Representative flow cytometry plots are shown together with the quantitative analysis. Data are presented as mean ± SD from three independent experiments (*n* = 3). Statistical differences among groups were analyzed using the Kruskal–Wallis test followed by pairwise comparisons with Bonferroni correction.

The survival rate was 83.71% in the Aβ-C (Aβ_1–42_-treated) group (Figure 3). Following GL treatment, the survival rates were 79.4%, 91.3%, 84.3%, 72.3%, and 79.5% at concentrations of 500, 250, 125, 62.5, and 31.25 µg/mL, respectively. The concentration-dependent antiapoptotic effect of GL extract observed by Annexin V analysis is further supported by the favorable docking interactions of both triterpenoids with human caspase-3 (PDB: 2XYG; −8.5 kcal/mol for Ganoderic Acid A and −7.8 kcal/mol for Ganoderenic Acid B) [20], suggesting a direct molecular basis for the observed inhibition of caspase-3-mediated apoptosis.

### 3.5. Mitochondrial Membrane Potential

**Figure 4:**
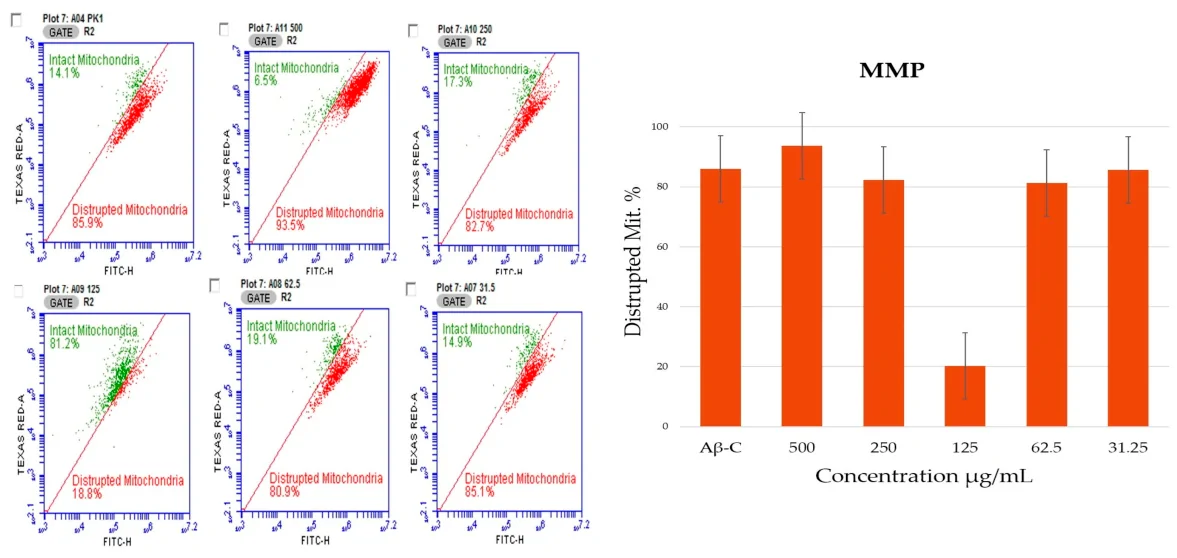
Mitochondrial membrane potential (ΔΨm) in Aβ_1–42_-treated SH-SY5Y cells following treatment with GL extract (31.25–500 µg/mL) for 24 h, determined using the JC-1 assay. Representative flow cytometry plots are shown together with the quantitative analysis of cells with disrupted mitochondrial membrane potential. Data are presented as mean ± SD from three independent experiments (*n* = 3). Statistical differences among groups were analyzed using the Kruskal–Wallis test followed by pairwise comparisons with Bonferroni correction.

The percentage of cells with disrupted mitochondrial membrane potential was 85.9% in the Aβ-C (Aβ_1–42_-treated) group (Figure 4). Following GL treatment, the percentages of cells with disrupted mitochondrial membrane potential were 93.7%, 82.3%, 20.3%, 81.3%, and 85.6% at concentrations of 500, 250, 125, 62.5, and 31.25 µg/mL, respectively. The preservation of mitochondrial membrane potential observed at moderate GL concentrations is consistent with the favorable binding affinities of Ganoderic Acid A (−9.2 kcal/mol) and Ganoderenic Acid B (−8.6 kcal/mol) toward human Bcl-2 (PDB: 4AQ3) [21], supporting a mechanism involving direct modulation of this antiapoptotic regulator.

### 3.6. ROS

**Figure 5:**
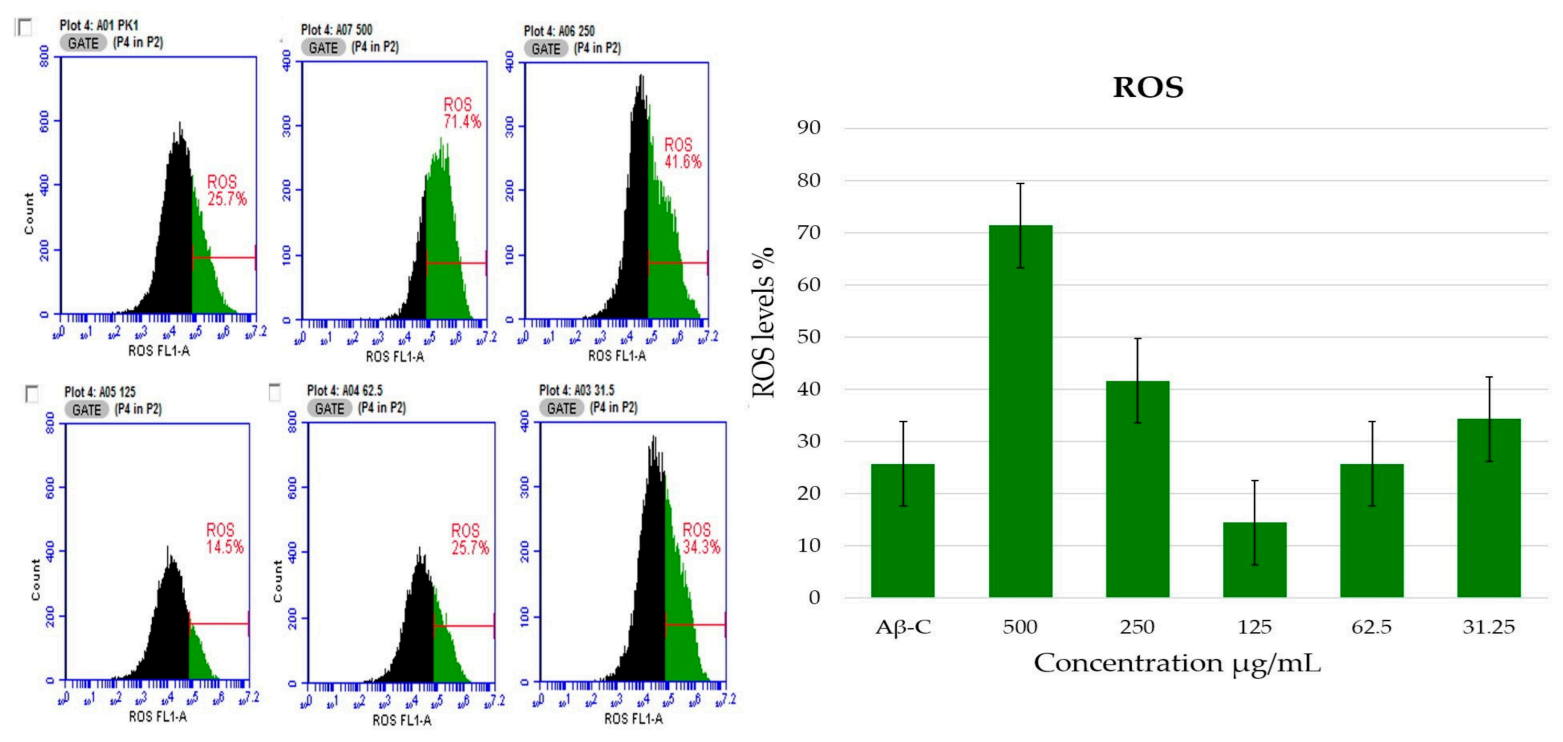
Intracellular ROS levels in Aβ_1–42_-treated SH-SY5Y cells following treatment with GL extract (31.25–500 µg/mL) for 24 h, determined using the DCF-DA assay. Representative flow cytometry histograms are shown together with the quantitative analysis. Data are presented as mean ± SD from three independent experiments (*n* = 3). Statistical differences among groups were analyzed using the Kruskal–Wallis test followed by pairwise comparisons with Bonferroni correction.

ROS levels in the Aβ-C (Aβ_1–42_-treated) group were 38.6% (Figure 5). Following 24 h of GL extract treatment, ROS levels were 71.4%, 41.6%, 14.5%, 25.7%, and 34.3% at concentrations of 500, 250, 125, 62.5, and 31.25 µg/mL, respectively. The non-linear modulation of ROS levels observed in this study may be related to the predicted binding affinities of Ganoderic Acid A (−10.4 kcal/mol) and Ganoderenic Acid B (−10.1 kcal/mol) toward the Keap1 Kelch domain (PDB: 4IN4) [22], a key regulator of the Nrf2 antioxidant response pathway. These computational findings suggest a potential relationship with redox-regulatory mechanisms.

### 3.7. Analysis of mRNA and Protein Levels of Cytokines

#### 3.7.1. TNF-α

**Figure 6:**
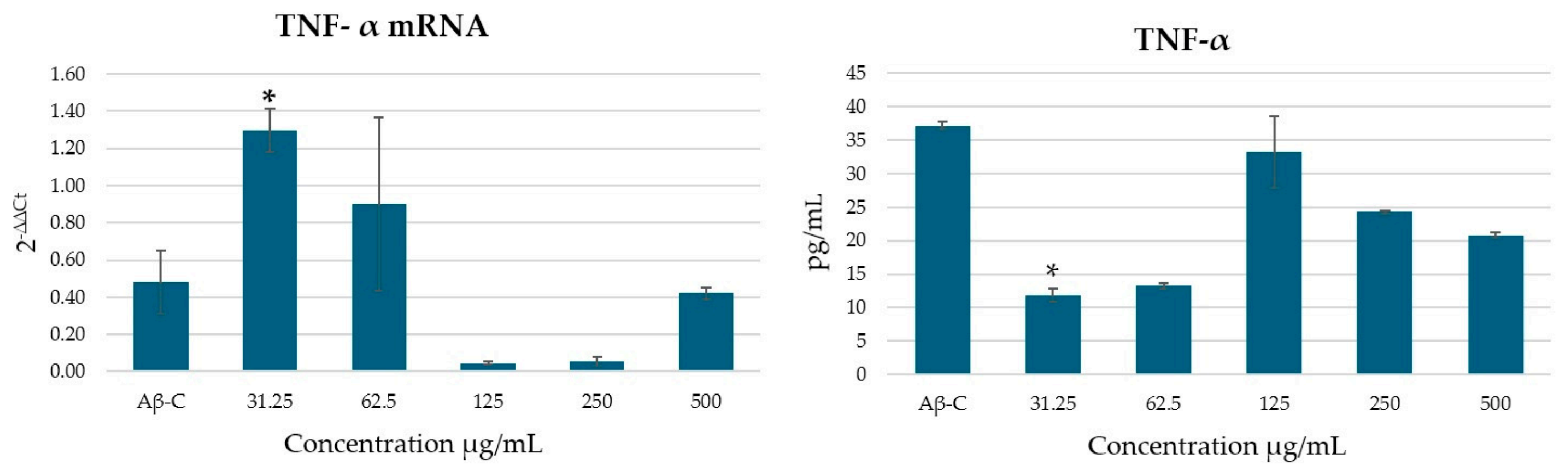
TNF-α mRNA expression and protein levels in Aβ_1–42_-treated SH-SY5Y cells following treatment with GL extract (31.25–500 µg/mL) for 24 h. Data are presented as mean ± SD from three independent experiments (*n* = 3). Statistical differences among groups were analyzed using the Kruskal–Wallis test followed by pairwise comparisons with Bonferroni correction. * *p* < 0.05 for mRNA expression and * *p* < 0.01 for protein levels compared with the Aβ-control group.

TNF-α mRNA levels differed significantly between groups when distributions were examined using the Kruskal–Wallis test (χ^2^ = 17.841, *p* < 0.007). The concentrations of 125 µg/mL and 31.5 µg/mL differed significantly from the control group, which was set at 1.00 (*p* < 0.05). TNF-α protein levels differed significantly among the groups (χ^2^ = 19.186, *p* < 0.004). Compared with the Aβ-C, 31.25 µg/mL showed a statistically significant increase (*p* < 0.01) (Figure 6). The observed reduction in TNF-α mRNA and protein expression following GL treatment is computationally supported by the favorable docking affinities of Ganoderic Acid A (−7.5 kcal/mol) and Ganoderenic Acid B (−7.8 kcal/mol) toward human TNF-α (PDB: 2AZ5) [17], lending direct molecular support to the observed anti-inflammatory effect.

#### 3.7.2. COX-2

**Figure 7:**
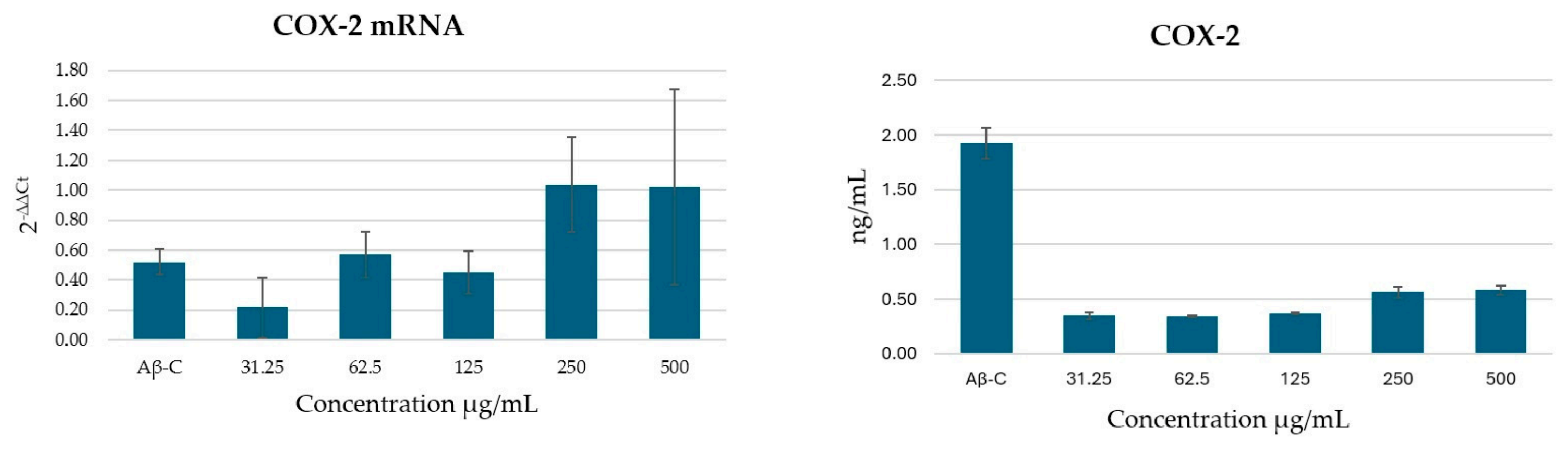
COX-2 mRNA expression and protein levels in Aβ_1–42_-treated SH-SY5Y cells following treatment with GL extract (31.25–500 µg/mL) for 24 h. Data are presented as mean ± SD from three independent experiments (*n* = 3). Statistical differences among groups were analyzed using the Kruskal–Wallis test followed by pairwise comparisons with Bonferroni correction.

COX-2 mRNA levels differed significantly between groups according to the Kruskal–Wallis test (χ^2^ = 13.807, *p* = 0.032); however, no significant pairwise differences were observed among the GL treatment groups (Figure 7). COX-2 protein levels also differed significantly among the groups (χ^2^ = 18.536, *p* = 0.005) (Figure 7). The reduction in COX-2 protein levels following GL treatment is consistent with the strong predicted binding affinities of Ganoderic Acid A (−10.1 kcal/mol) and Ganoderenic Acid B (−10.2 kcal/mol) for human COX-2 (PDB: 5F19), within the vicinity of the Ser530 catalytic acetylation site [19], providing structural support for the observed anti-inflammatory effect.

#### 3.7.3. IL-6

**Figure 8:**
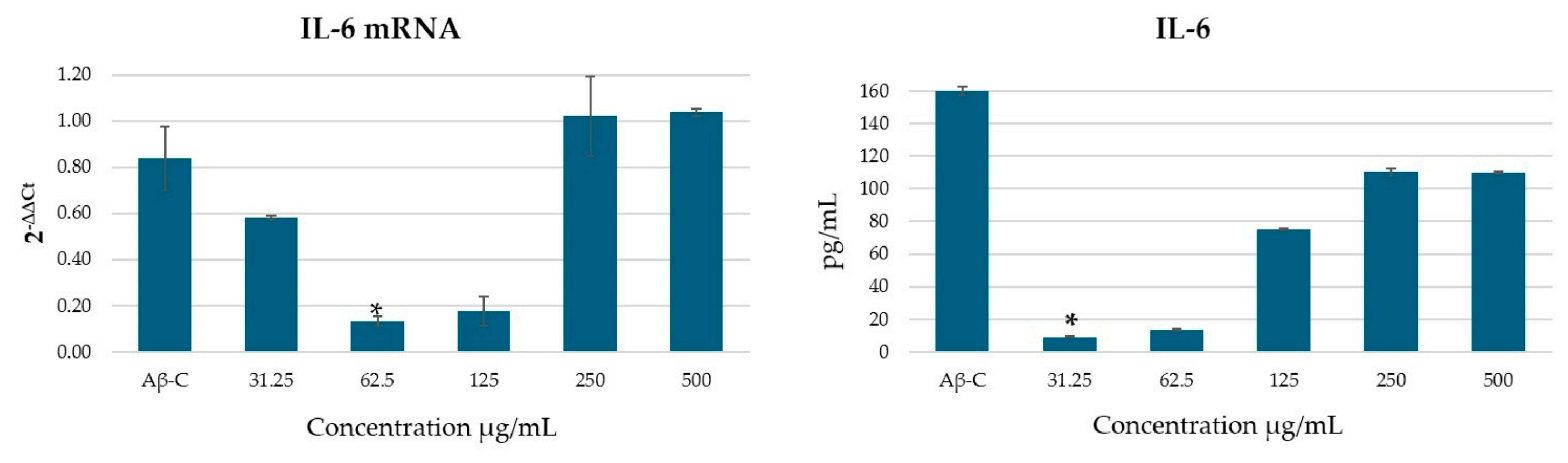
IL-6 mRNA expression and protein levels in Aβ_1–42_-treated SH-SY5Y cells following treatment with GL extract (31.25–500 µg/mL) for 24 h. Data are presented as mean ± SD from three independent experiments (*n* = 3). Statistical differences among groups were analyzed using the Kruskal–Wallis test followed by pairwise comparisons with Bonferroni correction. * *p* < 0.05 for mRNA expression and * *p* < 0.01 for protein levels compared with the Aβ-control group.

IL-6 mRNA levels differed significantly between groups when distributions were examined using the Kruskal–Wallis test (χ^2^ = 18.079, *p* < 0.006). The concentrations of 500 µg/mL and 62.5 µg/mL showed a significant difference relative to the control group, which was set to 1.00 (*p* < 0.05) (Figure 8). IL-6 protein levels differed significantly among the groups (χ^2^ = 19.290, *p* < 0.004). Compared with the control group, the 31.25 µg/mL and NC groups showed a statistically significant change (*p* < 0.01) (Figure 8). Consistent with these results, docking analysis revealed comparable and favorable binding of both Ganoderic Acid A and Ganoderenic Acid B to human IL-6 (PDB: 1ALU; −7.3 kcal/mol for both compounds) [18], lending computational support to the observed downregulation of IL-6 mRNA and protein expression.

#### 3.7.4. IL-10

**Figure 9:**
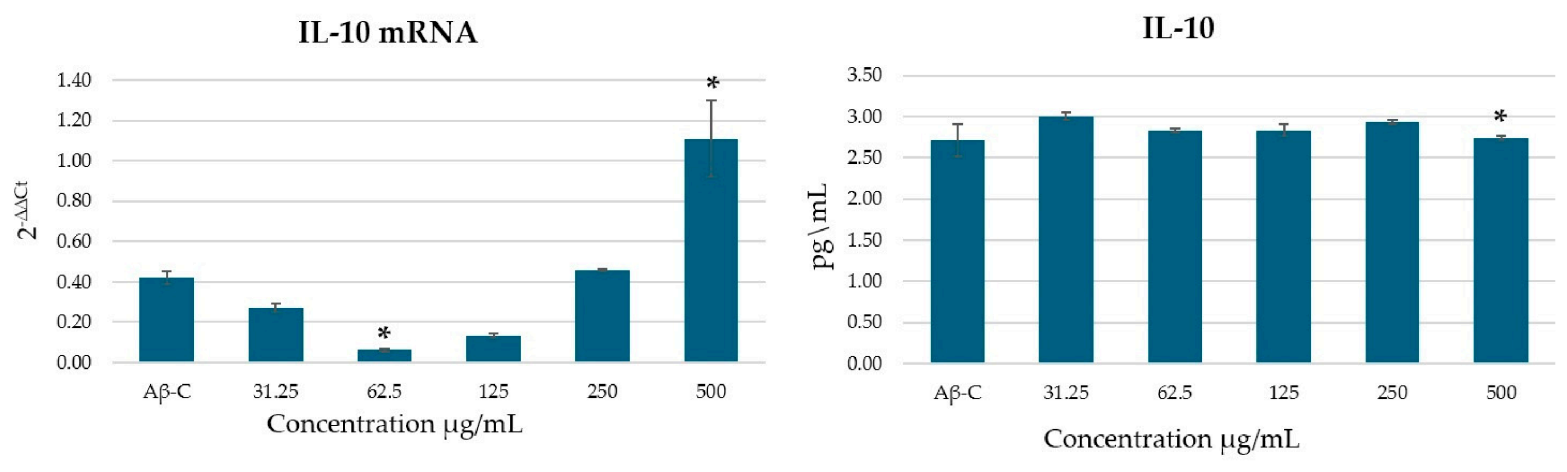
IL-10 mRNA expression and protein levels in Aβ_1–42_-treated SH-SY5Y cells following treatment with GL extract (31.25–500 µg/mL) for 24 h. Data are presented as mean ± SD from three independent experiments (*n* = 3). Statistical differences among groups were analyzed using the Kruskal–Wallis test followed by pairwise comparisons with Bonferroni correction. * *p* < 0.05 for mRNA expression and * *p* < 0.05 for protein levels compared with the Aβ-control group.

IL-10 mRNA levels differed significantly between groups when distributions were examined using the Kruskal–Wallis test (χ^2^ = 19.236, *p* < 0.004). The concentrations of 500 µg/mL and 62.5 µg/mL showed a significant difference relative to the Aβ-C group, which was set to 1.00 (*p* < 0.05) (Figure 9). IL-10 protein levels differed significantly among the groups (χ^2^ = 13.808, *p* < 0.032). Compared with the Aβ-C group, the 500 µg/mL and 31.25 µg/mL groups exhibited a statistically significant change (*p* < 0.05) (Figure 9).

#### 3.7.5. TGF-β1

**Figure 10:**
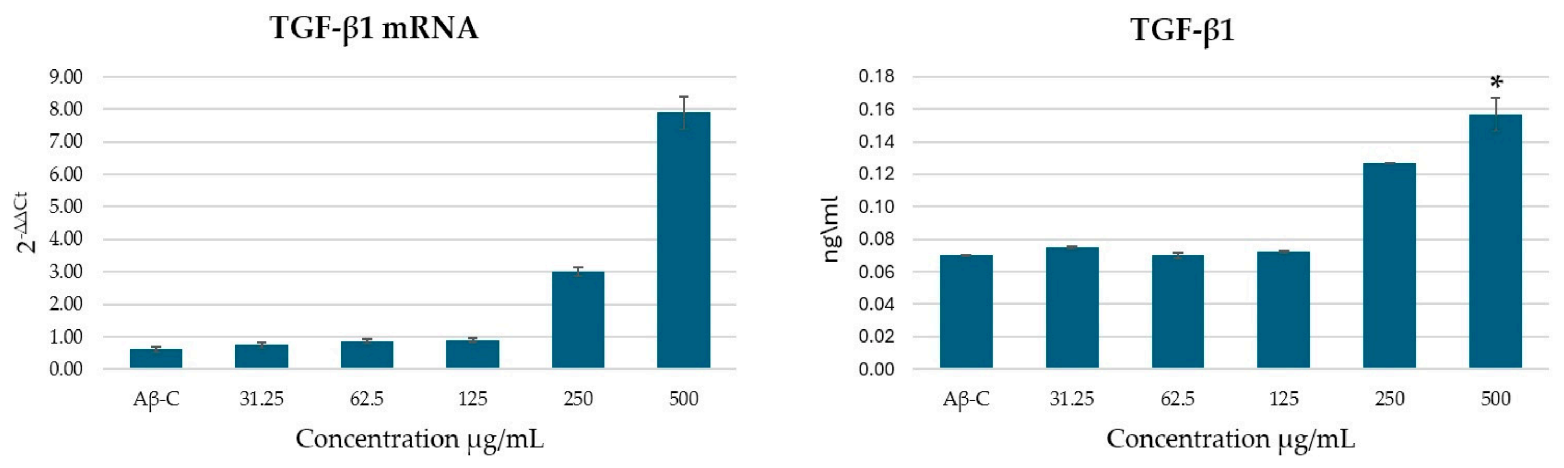
TGF-β1 mRNA expression and protein levels in Aβ_1–42_-treated SH-SY5Y cells following treatment with GL extract (31.25–500 µg/mL) for 24 h. Data are presented as mean ± SD from three independent experiments (*n* = 3). Statistical differences among groups were analyzed using the Kruskal–Wallis test followed by pairwise comparisons with Bonferroni correction. * *p* < 0.01 compared with the Aβ-C group.

TGF-β1 mRNA levels differed significantly between groups when distributions were examined using the Kruskal–Wallis test (χ^2^ = 19.028, *p* < 0.004). Differences in concentrations were not statistically significant. TGF-β1 protein levels differed significantly among the groups (χ^2^ = 19.248, *p* < 0.004). Compared with the Aβ-C group, the 500 µg/mL GL-treated group exhibited a statistically significant change (*p* < 0.01) (Figure 10).

Spearman’s rank correlation analysis demonstrated significant positive correlations between IL-6 mRNA expression and *COX-2*, *IL-10*, and *TGF-β1* (all *p* < 0.001). *COX-2* expression was also positively correlated with IL-10 and *TGF-β1* (all *p* < 0.001). In addition, a significant positive correlation was observed between *IL-10* and *TGF-β1* (*p* < 0.001) (Table 3).

Spearman’s rank correlation analysis of protein levels revealed significant positive correlations between AChE and IL-6 and AChE and TNF-α (both *p* < 0.001), and between COX-2 and TGF-β1 (*p* < 0.05). In addition, IL-6 protein levels were positively correlated with TNF-α (*p* < 0.001). Significant negative correlations were observed between IL-6 and IL-10 (*p* < 0.05) and between TNF-α and TGF-β1 protein levels (*p* < 0.05) (Table 4).

## 4. Discussion

The neuroprotective, anti-inflammatory, antiapoptotic, and antioxidant properties of GL have long been recognized in traditional medicine and reported at the phenomenological level in numerous pharmacological studies. However, the direct physical interactions driving these biological outcomes have lacked systematic structural characterization. Similarly, a recent study integrating experimental and computational approaches demonstrated the anti-Alzheimer potential of GL in a *C. elegans* model [32]; however, unlike the present study, its computational analyses focused primarily on direct interactions with Aβ rather than on multiple human protein targets associated with AD-related cellular mechanisms. In the present study, in silico molecular docking, ADME, and toxicity analyses were performed on Ganoderic Acid A and Ganoderenic Acid B against selected AD-related protein targets. The USP certificate confirms that both compounds are among the triterpenoid constituents of the standardized extract used in this study, supporting their selection for the docking analysis. Although the USP certificate does not provide their individual amounts in the extract, the docking results indicate that these compounds may interact with the selected targets. These findings were considered alongside the in vitro results for the corresponding cellular processes, including cholinergic dysfunction, inflammation, apoptosis, oxidative stress, and mitochondrial impairment, in Aβ_1–42_-induced SH-SY5Y cells.

GL has a long history of use in China and Japan as a medicinal mushroom, associated with benefits such as longevity, enhanced mental clarity, and increased disease resistance. In addition to this traditional “neurotonic” and “anti-aging” reputation, modern pharmacological research has shown that the triterpenoid and polysaccharide components of GL may target key mechanisms of Alzheimer’s pathology, including oxidative stress, neuroinflammation, and β-amyloid accumulation. Undifferentiated SH-SY5Y cells were used in the present study to establish an Aβ_1–42_-induced cellular model and evaluate the cellular response to Aβ exposure. SH-SY5Y cells exposed to Aβ have been widely used as an in vitro model to investigate AD-related cellular alterations and potential neuroprotective effects [24,25,26,27,28]. In our experimental design, the increase in AChE activity following Aβ_1–42_ exposure was considered one of the functional indicators supporting model establishment. Although undifferentiated SH-SY5Y cells retain proliferative and neuroblastoma-like characteristics, they provide a useful in vitro model for investigating cellular responses to Aβ_1–42_-induced toxicity. Further studies using differentiated neuronal models may help extend these findings to more mature neuronal systems [24,25,26,28].

Administration of acetylcholinesterase inhibitors improves cognition in patients with AD through increased acetylcholine availability in the brain. GL has also been reported to exhibit acetylcholinesterase-inhibitory activity, suggesting its potential use in AD treatment [33,34].

In our study, a decrease was observed across all concentrations in GL-applied cells compared to the Aβ-C, with a statistically significant reduction at 31.25 µg/mL (*p* < 0.01). GL and some of its constituents have also been reported to exhibit acetylcholinesterase-inhibitory effects, suggesting a potential cholinergic mechanism relevant to AD [35,36,37].

Treatment with *Ganoderma* spore oil, triterpenes, polysaccharides, and antioxidant peptides decreased oxidative stress, inhibited inflammation, and alleviated pain-induced cognitive impairment [38,39,40,41]. Studies indicate that it can inhibit critical inflammatory signaling pathways, including JAK/STAT, PI3K/Akt-NRF2, and TLR-4/NF-κB [36,41]. Our findings indicate that GL reduced the protein and mRNA expression of COX-2, IL-6, and TNF-α at all concentrations. In our study, we observed that COX-2 mRNA is downregulated, whereas COX-2 protein is upregulated, in the Aβ group; this mRNA–protein discrepancy may reflect post-translational modifications that independently regulate protein levels. The COX-2 protein may be influenced by increased enzymatic activity or stability in the presence of Aβ. Additionally, Aβ may activate signaling pathways that trigger cellular stress responses and inflammation, thereby increasing COX-2 protein levels. Wu et al.’s study showed that COX-2 and IL-6 expression levels decreased in a dose-dependent way [40]. Another study demonstrated that GL polysaccharides significantly suppressed Aβ42-induced proinflammatory cytokines, including IL-1β, IL-6, and iNOS, restoring their levels closer to baseline [42].

IL-10 is an important anti-inflammatory cytokine that modulates glial activation, thereby preventing inflammation-induced neuronal degeneration under pathological conditions. TGF-β1 is a common cytokine reported to have immunomodulatory and neuroprotective effects in the brain. According to our results, when the Aβ-C group and the extract-applied groups were compared, an increase was observed at 500 µg/mL. In contrast, IL-10 mRNA and protein expression increased at 500 µg/mL, whereas TGF-β1 mRNA and protein levels increased at 500 and 250 µg/mL. Studies in mice with chronic cerebral hyperplasia have demonstrated that GL polysaccharides alleviate cognitive impairment by increasing regulatory T cell (CD4^+^CD25^+^Foxp3^+^) populations, enhancing IL-10 and TGF-β1 levels, and modulating abnormal energy metabolism [43].

ROS are a major factor in the cellular damage linked to neurodegenerative diseases and aging. In AD, studies have demonstrated that ROS-induced accumulation of Aβ peptides disrupts the lysosomal membrane, ultimately contributing to neuronal death [44,45]. A progressive decline in mitochondrial function, a primary source of ROS generation, is another feature of both aging and AD. Furthermore, mitochondria are extremely vulnerable to oxidative damage [46]. Numerous studies have highlighted mitochondrial dysfunction and oxidative stress as fundamental events in the pathogenesis of AD [47,48].

In our study, intracellular ROS levels in Aβ_1–42_-exposed SH-SY5Y cells treated with GL extract exhibited a non-linear, biphasic response. Moderate extract concentrations (62.5–125 μg/mL) markedly reduced ROS production, whereas higher concentrations (250–500 μg/mL) led to a partial increase in ROS levels, indicating that the extract does not exert a strictly dose-dependent antioxidant effect across the entire concentration range. The nonlinear dose–response obtained for ROS levels may reflect the biphasic biological response of the GL extract. While moderate concentrations reduced oxidative stress, higher concentrations increased ROS production. This situation has been described for various natural products and can be attributed to concentration-dependent hormetic effects: low to moderate doses exhibit antioxidant activity, while higher doses may cause mild cellular stress or exhibit pro-oxidant properties. Our findings support selecting 125 µg/mL as the most biologically effective dose under experimental conditions. In contrast, TNF-α expression showed a more consistent downregulation within the same concentration range, suggesting differential regulation of oxidative stress and inflammatory pathways. This divergence likely reflects distinct cellular mechanisms governing redox homeostasis and cytokine signaling, highlighting the extract’s complex, multi-target mode of action.

Enhancing mitochondrial function is considered a potential therapeutic strategy to mitigate AD. Studies have reported that GL improves the pathologies of neurodegenerative diseases by regulating mitochondrial function and apoptosis [45,49,50]. Therefore, in our study, mitochondrial MMP, apoptosis, and ROS levels were assessed. Our results showed that MMP impairment decreased at all concentrations relative to ROS levels with Aβ-C, and at all concentrations except 500 and 250 µg/mL relative to Aβ-C. It is thought that high concentrations of extracts increase mitochondrial disruption and ROS levels in AD model cells. Apoptosis plays an important role in maintaining tissue homeostasis; however, aberrant activation of apoptotic pathways contributes to neuronal loss in neurodegenerative diseases, including AD [51]. The antiapoptotic activity of GL is due to ROS reduction and mitochondrial protection. GL triterpenoids have been shown to attenuate apoptosis and reduce the expression of apoptosis-related proteins in hippocampal tissues and neurons, thereby providing neuroprotection in AD mouse models [52]. Another study showed that the GL triterpenoid Ganoderic acid A (GAA) dose-dependently increased the viability of HT22 cells modeled with Aβ25-35 AD and inhibited the expression levels of proteins involved in the MAPK signaling pathway [53]. GAA also down-regulated cleaved caspase-3 protein levels and reduced apoptosis by inhibiting ERK signaling [54]. As a result, it was determined that GAA inhibited apoptosis via the ERK/MAPK signaling pathway. The antiapoptotic effect of GL is attributed to the reduction in reactive oxygen species and the protection of mitochondria. In our study, we observed that cell viability decreased at other doses, except for GL at 250 µg/mL and 125 µg/mL, compared to the Aβ-C. Our study findings indicate that dosage is a pivotal factor in GL extract administration, with excessive dosing during treatment potentially yielding deleterious rather than beneficial outcomes.

Taken together, the computational predictions and in vitro findings provide complementary evidence for a potential multi-target mechanism underlying the biological effects of GL. However, translating these promising cellular and structural findings into clinical efficacy must account for specific pharmacokinetic limitations. SwissADME analysis predicted low gastrointestinal absorption and lack of blood–brain barrier (BBB) permeability for both GAA and GAB. These predicted pharmacokinetic limitations should be considered when interpreting their potential relevance to AD and require further experimental validation. Furthermore, while ProTox-3.0 predictions affirmed a safe overall profile (LD50: 9000 mg/kg; Toxicity Class 6), the distinct immunotoxicity signal computationally identified for Ganoderenic Acid B—absent in Ganoderic Acid A—highlights the critical need for comprehensive in vivo toxicological and pharmacokinetic validation of these isolated compounds in future preclinical stages.

## 5. Conclusions

Overall, the multifactorial nature and complex pathology of AD render it an incurable disease, posing considerable challenges for patients, their families, and healthcare systems. This complexity continues to hinder the development of effective therapeutic interventions. However, with growing interest in natural compounds, GL has gained attention as a promising neuroprotective candidate. The bioactive compounds of GL have been shown to alleviate neuroinflammation, reduce neuronal apoptosis, improve mitochondrial function, and regulate ROS levels. While investigations into the potential of GL as a treatment for AD are ongoing, growing evidence substantiates its neuroprotective and beneficial roles. Evidence indicates that various GL components, including triterpenoids (e.g., ganosidone A), phenolic compounds (e.g., quercetin and ursolic acid), polysaccharides, and sterols, exert anti-inflammatory effects that may be advantageous in neurodegenerative disorders, particularly AD [22,25]. According to these outcomes, our study revealed that GL treatment mitigates inflammation by regulating gene transcription in an AD model. Additionally, GL exhibited neuroprotective actions by preserving mitochondrial membrane potential, limiting ROS accumulation, and suppressing neuronal apoptosis, supporting its potential as an adjunct therapeutic candidate for AD. Although in vitro models offer valuable mechanistic insights into neuronal responses under controlled conditions, applying these findings to in vivo settings is inherently limited by factors such as gastrointestinal biotransformation, systemic bioavailability, and blood–brain barrier permeability. Orally administered extracts may undergo metabolic changes, potentially altering their composition and effective concentrations in target brain regions compared with those applied directly to neuronal cells in vitro. Nonetheless, the present study was designed to investigate the direct cellular and molecular neuroprotective mechanisms of a standardized *Ganoderma lucidum* extract. However, further in vivo and pharmacokinetic studies are necessary to fully validate the clinical significance of the observed in vitro effects.

## Figures and Tables

**Table 1:** The primer sequences used in the RT-qPCR.

Gene	Primer Sequences (5′-3′)
*TNF-α*	F: AAAACAACCCTCAGACGCCA
*TNF-α*	R: TCCTTTCCAGGGGAGAGAGG
*TGF-β1*	F: ACCTGCCACAGATCCCCTAT
*TGF-β1*	R: GAGCAACACGGGTTCAGGTA
*IL-6*	F: CTTCGGTCCAGTTGCCTTCTC
*IL-6*	R: GGCATTTGTGGTTGGGTCAG
*IL-10*	F: TGCAAAAGAAGGCATGCACA
*IL-10*	R: CGCCTTGATGTCTGGGTCTT
*COX-2 (PGES2)*	F: TTGCATTCTTTGCCCAGCAC
*COX-2 (PGES2)*	R: ACCGTAGATGCTCAGGGACT
*GAPDH*	F: TTTTGCGTCGCCAGCC
*GAPDH*	R: ATGGAATTTGCCATGGGTGGA

**Table 2:** Molecular docking, physicochemical, and toxicological profiles of Ganoderic Acid A and Ganoderenic Acid B against human protein targets corresponding to in vitro endpoints.

Protein Target	PDB ID	In Vitro Endpoint	GAA Vina Score (kcal/mol)	GAB Vina Score (kcal/mol)
AChE	4EY7	AChE level	−9.4	−9.0
TNF-α	2AZ5	TNF-α mRNA/protein	−7.5	−7.8
COX-2 (PTGS2)	5F19	COX-2 mRNA/protein	−10.1	−10.2
IL-6	1ALU	IL-6 mRNA/protein	−7.3	−7.3
Caspase-3	2XYG	Annexin V apoptosis assay	−8.5	−7.8
Bcl-2	4AQ3	Mitochondrial membrane potential	−9.2	−8.6
Keap1/Nrf2	4IN4	ROS levels	−10.4	−10.1

**Table 3:** Spearman’s rank correlations among mRNA expression levels across different GL concentrations.

mRNA	TNF-α	COX-2	IL-10	TGF-β1
IL-6	−0.082/0.723	0.656 **/0.001	0.925 **/0.000	0.577 **/0.006
TNF-α		−0.104–0.104/0.653	−0.071–0.071/0.760	−0.376/0.093
COX-2			0.559 **/0.008	0.598 **/0.004
IL-10				0.587 **/0.005

Data are presented as Spearman’s correlation coefficient (r_s_)/*p*-value. ** *p* < 0.01.

**Table 4:** Spearman’s rank correlations among protein levels across different GL concentrations.

Protein	IL-6	TNF-α	COX-2	IL-10	TGF-β1
AChE	0.931 **/0.000	0.861 **/0.000	0.337/0.135	−0.326–0.326/0.150	−0.410/0.065
IL-6		0.766 **/0.000	0.416/0.061	−0.473 */0.030	−0.284/0.212
TNF-α			0.033/0.887	−0.295/0.194	−0.511 */0.018
COX-2				−0.375/0.094	0.462 */0.035
IL-10					0.079/0.733

Data are presented as Spearman’s correlation coefficient (r_s_)/*p*-value. * *p* < 0.05; ** *p* < 0.01.

## Data Availability

The data presented in this study are available on reasonable request from the corresponding author. The data are not publicly available due to institutional restrictions.

## Supplementary Materials

The following supporting information can be downloaded at https://www.mdpi.com/article/10.3390/molecules31183172/s1, File S1: USP Certificate for Ganoderma lucidum Fruiting Body Dry Extract (Catalog No. 1288372, Lot No. F012B0), including the typical chromatogram provided by USP; Table S1: Complete CB-Dock2 blind docking results; Table S2: Full ProTox-3.0 toxicological profile of Ganoderic Acid A and Ganoderenic Acid B. References [30,31] are cited in the Supplementary Materials.

## Abbreviations

The following abbreviations are used in this manuscript:

AChE	Acetylcholinesterase
AD	Alzheimer’s disease
COX-2	Cyclooxygenase-2
DMEM	Dulbecco’s Modified Eagle Medium
FBS	Fetal bovine serum
GAA	Ganoderic acid A
GL	*Ganoderma lucidum*
IL-10	Interleukin 10
IL-6	Interleukin 6
JAK/STAT	Janus kinase/signal transducers and activators of transcription
MMP	Mitochondrial Membrane Potential
C	Control
NOS	Nitric Oxide Synthase
NRF2	Nuclear Erythroid 2-Related Factor 2
Aβ-C	Aβ-Control
PI3K/Akt-	Phosphatidylinositol 3-Kinase/Akt pathway
ROS	Reactive Oxygen Species
TGF-β1	Transforming Growth Factor Beta 1
TLR-4/NF-κB	Toll-Like Receptor 4/Nuclear Factor Kappa B
TNF-α	Tumor Necrosis Factor Alpha

## References

[1] Jorfi M., Maaser-Hecker A., Tanzi R.E. (2023). The neuroimmune axis of Alzheimer’s disease. Genome Med..

[2] Duan Y., Han C., Zheng H., Yu J., Luo M. (2025). Global, regional, and national burden of Alzheimer’s disease and other dementias from 1990 to 2021: Findings from the Global Burden of Disease Study 2021. Front. Aging Neurosci..

[3] Li X., Feng X., Sun X., Hou N., Han F., Liu Y. (2022). Global, regional, and national burden of Alzheimer’s disease and other dementias, 1990–2019. Front. Aging Neurosci..

[4] Alzheimer’s Association (2024). 2024 Alzheimer’s disease facts and figures. Alzheimers Dement..

[5] Chen J., Lin B., Seow E., Kim S., Bajaj P.S., Mattke S. (2025). Prediction of infusion capacity to deliver Alzheimer’s disease treatments in the United States relative to expected demand. J. Alzheimers Dis..

[6] Ali J., Choe K., Park J.S., Park H.Y., Kang H., Park T.J., Kim M.O. (2024). The Interplay of Protein Aggregation, Genetics, and Oxidative Stress in Alzheimer’s Disease: Role for Natural Antioxidants and Immunotherapeutics. Antioxidants.

[7] Weinstock M. (2025). Role of Oxidative Stress and Neuroinflammation in the Etiology of Alzheimer’s Disease: Therapeutic Options. Antioxidants.

[8] Wójcik P., Jastrzębski M.K., Zięba A., Matosiuk D., Kaczor A.A. (2024). Caspases in Alzheimer’s Disease: Mechanism of Activation, Role, and Potential Treatment. Mol. Neurobiol..

[9] Zhang J., Zhang Y., Wang J., Xia Y., Zhang J., Chen L. (2024). Recent advances in Alzheimer’s disease: Mechanisms, clinical trials and new drug development strategies. Signal Transduct. Target. Ther..

[10] Firdous S.M., Khan S.A., Maity A. (2024). Oxidative stress-mediated neuroinflammation in Alzheimer’s disease. Naunyn Schmiedebergs Arch. Pharmacol..

[11] Adotey G., Quarcoo A., Gedel M.A., Yerenkyi P., Otu P., Anang A.K., Okine L.K.N., Gbewonyo W.S.K., Holliday J.C., Lombardi V.C. (2026). The Medicinal Mushroom Ganoderma: A Review of Systematics, Phylogeny, and Metabolomic Insights. J. Fungi.

[12] Ahmad M.F., Alsayegh A.A., Ahmad F.A., Akhtar M.S., Alavudeen S.S., Bantun F., Wahab S., Ahmed A., Ali M., Elbendary E.Y. (2024). *Ganoderma lucidum*: Insight into antimicrobial and antioxidant properties with development of secondary metabolites. Heliyon.

[13] Wachtel-Galor S., Yuen J., Buswell J.A., Benzie I.F.F., Benzie I.F.F., Wachtel-Galor S. (2011). *Ganoderma lucidum* (Lingzhi or Reishi): A Medicinal Mushroom. Herbal Medicine: Biomolecular and Clinical Aspects.

[14] Lian W., Yang X., Duan Q., Li J., Zhao Y., Yu C., He T., Sun T., Zhao Y., Wang W. (2024). The Biological Activity of *Ganoderma lucidum* on Neurodegenerative Diseases: The Interplay between Different Active Compounds and the Pathological Hallmarks. Molecules.

[15] Daina A., Michielin O., Zoete V. (2017). SwissADME: A free web tool to evaluate pharmacokinetics, drug-likeness and medicinal chemistry friendliness of small molecules. Sci. Rep..

[16] Cheung J., Rudolph M.J., Burshteyn F., Cassidy M.S., Gary E.N., Love J., Franklin M.C., Height J.J. (2012). Structures of human acetylcholinesterase in complex with pharmacologically important ligands. J. Med. Chem..

[17] He M.M., Smith A.S., Oslob J.D., Flanagan W.M., Braisted A.C., Whitty A., Cancilla M.T., Wang J., Lugovskoy A.A., Yoburn J.C. (2005). Small-molecule inhibition of TNF-alpha. Science.

[18] Somers W., Stahl M., Seehra J.S. (1997). 1.9 A crystal structure of interleukin 6: Implications for a novel mode of receptor dimerization and signaling. EMBO J..

[19] Lucido M.J., Orlando B.J., Vecchio A.J., Malkowski M.G. (2016). Crystal Structure of Aspirin-Acetylated Human Cyclooxygenase-2: Insight into the Formation of Products with Reversed Stereochemistry. Biochemistry.

[20] Ganesan R., Jelakovic S., Mittl P.R., Caflisch A., Grütter M.G. (2011). In silico identification and crystal structure validation of caspase-3 inhibitors without a P1 aspartic acid moiety. Acta Crystallogr. Sect. F Struct. Biol. Cryst. Commun..

[21] Perez H.L., Banfi P., Bertrand J., Cai Z.W., Grebinski J.W., Kim K., Lippy J., Modugno M., Naglich J., Schmidt R.J. (2012). Identification of a phenylacylsulfonamide series of dual Bcl-2/Bcl-xL antagonists. Bioorg. Med. Chem. Lett..

[22] Lo S.C., Li X., Henzl M.T., Beamer L.J., Hannink M. (2006). Structure of the Keap1:Nrf2 interface provides mechanistic insight into Nrf2 signaling. EMBO J..

[23] Yardim-Akaydin S., Elçi M.P., Fatsa T. (2025). Investigation of Pro-inflammatory and Anti-inflammatory Effects of Different Ω3/Ω6 PUFA Ratios on Alzheimer’s Model Induced with Amyloid β 1-42 in Human SHSY5Y Cells by In vitro Methods. Neurol. Sci. Neurophysiol..

[24] Wang P., Liao W., Fang J., Liu Q., Yao J., Hu M., Ding K. (2014). A glucan isolated from flowers of Lonicera japonica Thunb. inhibits aggregation and neurotoxicity of Aβ42. Carbohydr. Polym..

[25] Wei W., Wang X., Kusiak J.W. (2002). Signaling events in amyloid beta-peptide-induced neuronal death and insulin-like growth factor I protection. J. Biol. Chem..

[26] Xiong W.P., Yao W.Q., Wang B., Liu K. (2021). BMSCs-exosomes containing GDF-15 alleviated SH-SY5Y cell injury model of Alzheimer’s disease via AKT/GSK-3β/β-catenin. Brain Res. Bull..

[27] Wang X., Zhang M., Liu H. (2019). LncRNA17A regulates autophagy and apoptosis of SH-SY5Y cell line as an in vitro model for Alzheimer’s disease. Biosci. Biotechnol. Biochem..

[28] Tan M.A., Ishikawa H., An S.S.A. (2022). Pandanus amaryllifolius Exhibits In Vitro Anti-Amyloidogenic Activity and Promotes Neuroprotective Effects in Amyloid-β-Induced SH-SY5Y Cells. Nutrients.

[29] United States Pharmacopeia (2015). Ganoderma Lucidum Fruiting Body.

[30] Liu Y., Yang X., Gan J., Chen S., Xiao Z.X., Cao Y. (2022). CB-Dock2: Improved protein-ligand blind docking by integrating cavity detection, docking and homologous template fitting. Nucleic Acids Res..

[31] Banerjee P., Kemmler E., Dunkel M., Preissner R. (2024). ProTox 3.0: A webserver for the prediction of toxicity of chemicals. Nucleic Acids Res..

[32] Mia M., Dutta A., Hossain M.M., Adhikari J., Rahman M.M., Shibly A.Z. (2026). Exploring the neuroprotective, antioxidant, and anti-amyloid effects of *Ganoderma lucidum* compounds in Alzheimer’s disease: Insights from experimental and computational approaches. J. Genet. Eng. Biotechnol..

[33] Gao Y., Liu Y., Li Y. (2024). Safety and efficacy of acetylcholinesterase inhibitors for Alzheimer’s disease: A systematic review and meta-analysis. Adv. Clin. Exp. Med..

[34] Jiang Y., Gao H., Turdu G. (2017). Traditional Chinese medicinal herbs as potential AChE inhibitors for anti-Alzheimer’s disease: A review. Bioorg. Chem..

[35] Huang C.H., Liao Y.T., Chen C.L., Tsai G.J. (2024). Protective effect of *Ganoderma lucidum*-fermented crop extracts against hydrogen peroxide- or β-amyloid-induced damage in human neuronal SH-SY5Y cells. BMC Complement. Med. Ther..

[36] Hasnat A., Pervin M., Lim B.O. (2013). Acetylcholinesterase inhibition and in vitro and in vivo antioxidant activities of *Ganoderma lucidum* grown on germinated brown rice. Molecules.

[37] Luo Q., Yang Z.-L., Cheng Y.-X. (2019). Dayaolingzhiols A−E, AchE inhibitory meroterpenoids from *Ganoderma lucidum*. Tetrahedron.

[38] Zhao H.L., Cui S.Y., Qin Y., Liu Y.T., Cui X.Y., Hu X., Kurban N., Li M.Y., Li Z.H., Xu J. (2021). Prophylactic effects of sporoderm-removed *Ganoderma lucidum* spores in a rat model of streptozotocin-induced sporadic Alzheimer’s disease. J. Ethnopharmacol..

[39] Choi S., Nguyen V.T., Tae N., Lee S., Ryoo S., Min B.S., Lee J.H. (2014). Anti-inflammatory and heme oxygenase-1 inducing activities of lanostane triterpenes isolated from mushroom *Ganoderma lucidum* in RAW264.7 cells. Toxicol. Appl. Pharmacol..

[40] Wu Y.L., Han F., Luan S.S., Ai R., Zhang P., Li H., Chen L.X. (2019). Triterpenoids from *Ganoderma lucidum* and Their Potential Anti-inflammatory Effects. J. Agric. Food Chem..

[41] Krobthong S., Yingchutrakul Y. (2020). Identification and enhancement of antioxidant P1-peptide isolated from *Ganoderma lucidum* hydrolysate. Food Biotechnol..

[42] Cai Q., Li Y., Pei G. (2017). Polysaccharides from *Ganoderma lucidum* attenuate microglia-mediated neuroinflammation and modulate microglial phagocytosis and behavioural response. J. Neuroinflamm..

[43] Zhang Y., Song S., Li H., Wang X., Song L., Xue J. (2022). Polysaccharide from *Ganoderma lucidum* alleviates cognitive impairment in a mouse model of chronic cerebral hypoperfusion by regulating CD4(+)CD25(+)Foxp3(+) regulatory T cells. Food Funct..

[44] Chong Z.Z., Souayah N. (2025). Oxidative Stress: Pathological Driver in Chronic Neurodegenerative Diseases. Antioxidants.

[45] Yu N., Pasha M., Chua J.J.E. (2024). Redox changes and cellular senescence in Alzheimer’s disease. Redox Biol..

[46] Wang X., Wang W., Li L., Perry G., Lee H.G., Zhu X. (2014). Oxidative stress and mitochondrial dysfunction in Alzheimer’s disease. Biochim. Biophys. Acta.

[47] Moawad M., Serag I., Alkhawaldeh I.M., Abbas A., Sharaf A., Alsalah S., Sadeq M.A., Shalaby M.M.M., Hefnawy M.T., Abouzid M. (2025). Exploring the Mechanisms and Therapeutic Approaches of Mitochondrial Dysfunction in Alzheimer’s Disease: An Educational Literature Review. Mol. Neurobiol..

[48] Rahman M.A., Rahman M.D.H., Rhim H., Kim B. (2024). Drug Target to Alleviate Mitochondrial Dysfunctions in Alzheimer’s Disease: Recent Advances and Therapeutic Implications. Curr. Neuropharmacol..

[49] Guo S.S., Cui X.L., Rausch W.D. (2016). *Ganoderma lucidum* polysaccharides protect against MPP(+) and rotenone-induced apoptosis in primary dopaminergic cell cultures through inhibiting oxidative stress. Am. J. Neurodegener. Dis..

[50] Tobore T.O. (2019). On the central role of mitochondria dysfunction and oxidative stress in Alzheimer’s disease. Neurol. Sci..

[51] Thal D.R., Gawor K., Moonen S. (2024). Regulated cell death and its role in Alzheimer’s disease and amyotrophic lateral sclerosis. Acta Neuropathol..

[52] Ma F., Wang J., Jiang W., Luo J., Yang R., Zhang L., Han C. (2024). Ganoderic Acid A: A Potential Natural Neuroprotective Agent for Neurological Disorders: A Review. Int. J. Med. Mushrooms.

[53] Shao N., Lu Q., Ouyang Z., Yang P., Wei T., Wang J., Cai B. (2024). Ganoderic acid a alleviates Aβ(25-35)-induced HT22 cell apoptosis through the ERK/MAPK pathway: A system pharmacology and in vitro experimental validation. Metab. Brain Dis..

[54] Yu N., Huang Y., Jiang Y., Zou L., Liu X., Liu S., Chen F., Luo J., Zhu Y. (2020). *Ganoderma lucidum* Triterpenoids (GLTs) Reduce Neuronal Apoptosis via Inhibition of ROCK Signal Pathway in APP/PS1 Transgenic Alzheimer’s Disease Mice. Oxidative Med. Cell. Longev..

