# Supplementary material for: Therapeutic Effects of Ganoderma lucidum Against Aβ-Induced Neurotoxicity in SH-SY5Y Cells: In Vitro Evidence and Computational Validation of Human Protein Targets
Source: Molecules. 2026 Sep 9;31(18):3172. doi: 10.3390/molecules31183172 (PMC13609799; doi:10.3390/molecules31183172)
Supplement: Supplementary file 1 [file molecules-31-03172-s001.zip › molecules-4506117-supplementary File S1.pdf]

Certificate

GANODERMA LUCIDUM FRUITING BODY DRY EXTRACT

USP Catalog No.:	1288372
USP Lot No.:	F012B0

Only compendial components as listed in the monograph are shown below

A mixture which includes ganoderenic acid C, ganoderic acid C2, ganoderic acid G, ganoderenic acid B, ganoderic acid B, ganoderic acid A, ganoderic acid H, ganoderenic acid D, ganoderic acid D, ganoderic acid F, and polysaccharides	CAS No.	**NA**
	Molecular Formula	**NA**
	Molecular Weight	**NA**

Copyright 2014 The United States Pharmacopeial Convention. All rights reserved.

LABEL TEXT

For use with specified USP compendial tests. Not for use as a drug. See SDS prior to use at www.usp.org/sds.

USP REFERENCE STANDARD

GANODERMA LUCIDUM FRUITING BODY DRY EXTRACT 1 g

Do not dry. Keep container tightly closed. Protect from light and moisture. Store at controlled room temperature. This material is hygroscopic.

USP, 12601 Twinbrook Pkwy, Rockville, MD, +1-301-881-0666
CAT. NO. 1288372 Material mfd. in Canada

LOT: F012B0

Teri L. Toth

Quality Assurance

Calculation Value

Unless otherwise stated on the Reference Standard label, a value of 100.0% should be used in the compendial applications for which the use of this Reference Standard is intended. Please refer to the specific Reference Standard label for further information.

Expiration

Current lots are identified in the current USP Catalog. In some cases, the previous lot may still be considered valid for use. If so, it is identified in the column marked “Previous Lot/Valid Use Date.”

It is the responsibility of each user to determine that this lot is current or valid when used. For the most up-to-date information, please refer to the USP Store at www.usp.org.

Instructions for Use

Follow the instructions on the label of the USP Reference Standard and in the appropriate USP documentary standard(s).

Non-Monograph Use

The suitability of this Reference Standard for use in non-compendial applications is solely the responsibility of the user.

LEGAL NOTICE

USP WARRANTS GOOD TITLE TO USP REFERENCE STANDARDS ON DISPATCH FROM USP. THE FOREGOING WARRANTY IS IN LIEU OF ANY OTHER WARRANTIES, EXPRESS OR IMPLIED, INCLUDING WITHOUT LIMITATION ANY WARRANTY OF MERCHANTABILITY OR FITNESS FOR A PARTICULAR PURPOSE, OR ANY WARRANTY THAT THE PRODUCTS, INCLUDING THIS CERTIFICATE, ARE OF MERCHANTABLE QUALITY. USP’S LIABILITY ARISING OUT OF OR RELATING TO THE SUPPLY OF USP REFERENCE STANDARDS AND THIS CERTIFICATE SHALL IN NO EVENT INCLUDE LOSS OF PROFITS, COST OF PROCURING SUBSTITUTE GOODS OR SERVICES, OR ANY INCIDENTAL, INDIRECT, OR CONSEQUENTIAL DAMAGES OF ANY KIND, EVEN IF USP IS AWARE OF THE POSSIBILITY OF SUCH DAMAGES. WITHOUT LIMITING THE GENERALITY OF THE FOREGOING, USP DOES NOT WARRANT THAT THE USE OR RESALE OF USP REFERENCE STANDARDS, INCLUDING THEIR USE TO PERFORM TESTS AND ASSAYS PUBLISHED BY USP, WILL NOT INFRINGE UNITED STATES OR ANY OTHER PATENTS.

USP Reference Standards are not intended for use as drugs, dietary supplements, or as medical devices.

This certificate may not be reproduced without the express written permission of USP.

Copyright 2014 The United States Pharmacopeial Convention. All rights reserved.

Typical Chromatogram

USP GANODERMA LUCIDUM FRUITING BODY DRY EXTRACT

**Catalog Number:** 1288372	
**Lot:** F012B0	
**Monograph:** Ganoderma lucidum Fruiting Body Dry Extract	
**Publication:** Herbal Medicines Compendium	
**Test:** Identification B and Content of Triterpenoic Acids	
**Sample:** Standard Solution B	

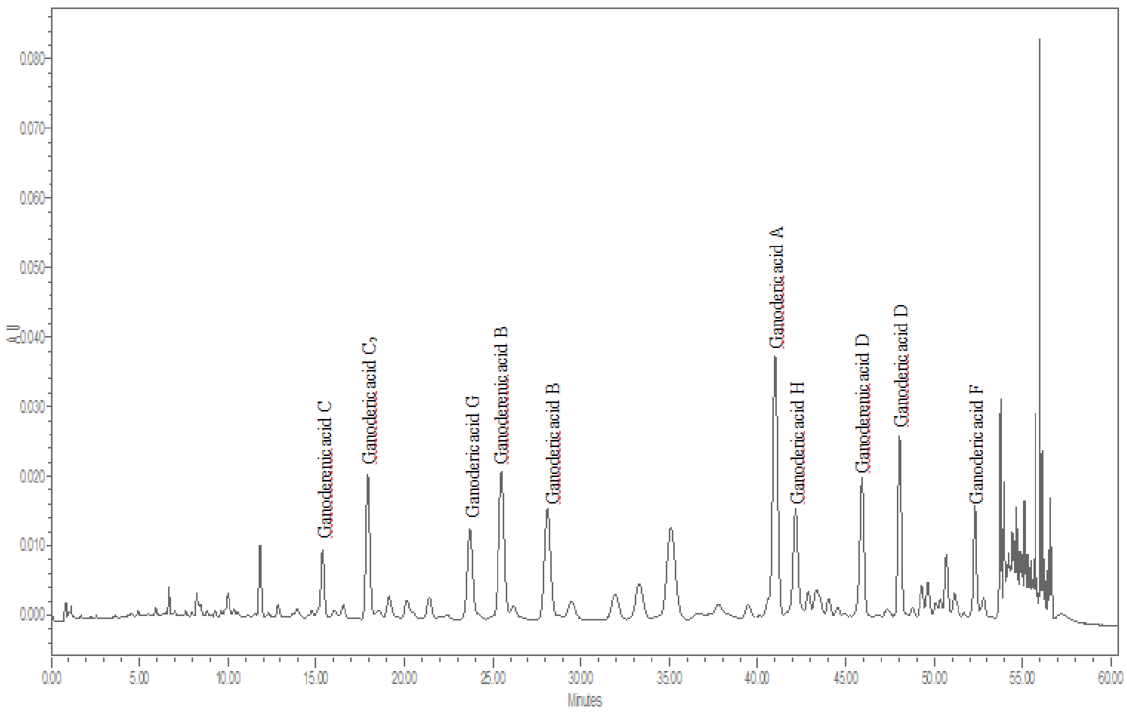

This chromatogram is supplied for information only, unless otherwise specified in an applicable monograph or general chapter.

Copyright 2014 The United States Pharmacopeial Convention. All rights reserved.
