# Supplementary material for: Therapeutic Effects of Ganoderma lucidum Against Aβ-Induced Neurotoxicity in SH-SY5Y Cells: In Vitro Evidence and Computational Validation of Human Protein Targets
Source: Molecules. 2026 Sep 9;31(18):3172. doi: 10.3390/molecules31183172 (PMC13609799; doi:10.3390/molecules31183172)
Supplement: Supplementary file 1 [file molecules-31-03172-s001.zip › molecules-4506117-supplementary tables.pdf]

Supplementary Materials

Table S1. Complete CB-Dock2 blind docking results for Ganoderic Acid A (GAA) and Ganoderenic Acid B (GAB) against human protein targets corresponding to the in vitro endpoints assessed in this study. For each target, all five top-ranked binding cavities identified by the CurPocket algorithm are reported, including Vina binding energy score, predicted cavity volume, docking box center coordinates, and docking box size. The cavity yielding the most favorable (most negative) Vina score for each target-ligand pair is presented in the main text (Table 2) and is indicated in below.

Target: Acetylcholinesterase (AChE; PDB: 4EY7)

Ganoderic Acid A

CurPocket ID	Vina score (kcal/mol)	Cavity volume (Å3)	Center (x, y, z)	Docking size (x, y, z)
1	-9.4	1410	10, -55, -25	25, 25, 25
2	-9.4	855	-14, -43, 27	25, 25, 25
3	-8.9	752	-7, -37, 14	25, 25, 25
4	-8.6	1259	-2, -50, -17	25, 25, 25
5	-7.7	1164	-8, -49, 45	25, 25, 25

Ganoderenic Acid B

CurPocket ID	Vina score (kcal/mol)	Cavity volume (Å3)	Center (x, y, z)	Docking size (x, y, z)
1	-9.0	1410	10, -55, -25	25, 25, 25
2	-8.9	752	-7, -37, 14	25, 25, 25
3	-8.8	1259	-2, -50, -17	25, 25, 25
4	-8.8	855	-14, -43, 27	25, 25, 25
5	-6.3	1164	-8, -49, 45	25, 25, 25

Target: Tumor Necrosis Factor-alpha (TNF-α; PDB: 2AZ5)

Ganoderic Acid A

CurPocket ID	Vina score (kcal/mol)	Cavity volume (Å3)	Center (x, y, z)	Docking size (x, y, z)
C2	-7.5	97	-23, 38, 20	25, 25, 25
C5	-7.1	54	-11, 42, 27	25, 25, 25
C3	-7.0	80	-21, 49, 16	25, 25, 25
C4	-6.1	69	-6, 40, 9	25, 25, 25
C1	-5.8	731	-11, 27, 25	25, 25, 25

Ganoderenic Acid B

CurPocket ID	Vina score (kcal/mol)	Cavity volume (Å3)	Center (x, y, z)	Docking size (x, y, z)
C2	-7.8	97	-23, 38, 20	23, 23, 23
C3	-6.6	80	-21, 49, 16	23, 23, 23
C4	-6.1	69	-6, 40, 9	23, 23, 23
C5	-5.7	54	-11, 42, 27	23, 23, 23
C1	-5.4	731	-11, 27, 25	23, 23, 23

Target: Cyclooxygenase-2 (COX-2; PDB: 5F19)

Ganoderic Acid A

CurPocket ID	Vina score (kcal/mol)	Cavity volume (Å3)	Center (x, y, z)	Docking size (x, y, z)
C2	-10.1	4431	14, 49, 65	25, 25, 25
C1	-9.7	26146	22, 39, 36	35, 35, 35
C5	-9.3	1167	31, 42, 38	25, 25, 25
C3	-8.4	1234	32, 37, 18	25, 25, 32
C4	-8.0	1233	28, 28, 59	25, 25, 33

Ganoderenic Acid B

CurPocket ID	Vina score (kcal/mol)	Cavity volume (Å3)	Center (x, y, z)	Docking size (x, y, z)
C5	-10.2	1167	31, 42, 38	23, 23, 23
C1	-9.5	26146	22, 39, 36	35, 35, 35
C4	-8.6	1233	28, 28, 59	23, 23, 33
C2	-7.8	4431	14, 49, 65	23, 29, 29
C3	-7.5	1234	32, 37, 18	23, 23, 32

Target: Interleukin-6 (IL-6; PDB: 1ALU)

Ganoderic Acid A

CurPocket ID	Vina score (kcal/mol)	Cavity volume (Å3)	Center (x, y, z)	Docking size (x, y, z)
C1	-7.3	722	6, -24, 18	25, 25, 25
C4	-6.7	166	14, -31, 0	25, 25, 25
C5	-6.6	134	0, -10, 23	25, 25, 25
C2	-5.9	436	-5, -27, 7	25, 25, 25
C3	-5.9	218	0, -23, -2	25, 25, 25

Ganoderenic Acid B

CurPocket ID	Vina score (kcal/mol)	Cavity volume (Å3)	Center (x, y, z)	Docking size (x, y, z)
C4	-7.3	166	14, -31, 0	23, 23, 23
C1	-7.1	722	6, -24, 18	23, 23, 23
C2	-6.4	436	-5, -27, 7	23, 23, 23
C5	-6.4	134	0, -10, 23	23, 23, 23
C3	-6.1	218	0, -23, -2	23, 23, 23

Target: Caspase-3 (PDB: 2XYG)

Ganoderic Acid A

CurPocket ID	Vina score (kcal/mol)	Cavity volume (Å3)	Center (x, y, z)	Docking size (x, y, z)
C1	-8.5	301	23, 9, 33	25, 25, 25
C5	-8.1	132	43, 26, 26	25, 25, 25
C4	-7.5	145	37, 33, 29	25, 25, 25
C2	-5.9	221	27, 30, 47	25, 25, 25
C3	-5.6	173	14, 32, 45	25, 25, 25

Ganoderenic Acid B

CurPocket ID	Vina score (kcal/mol)	Cavity volume (Å3)	Center (x, y, z)	Docking size (x, y, z)
C4	-7.8	145	37, 33, 29	23, 23, 23
C5	-7.1	132	43, 26, 26	23, 23, 23
C1	-6.3	301	23, 9, 33	23, 23, 23
C3	-5.5	173	14, 32, 45	23, 23, 23
C2	-5.4	221	27, 30, 47	23, 23, 23

Target: B-cell Lymphoma 2 (Bcl-2; PDB: 4AQ3)

Ganoderic Acid A

CurPocket ID	Vina score (kcal/mol)	Cavity volume (Å3)	Center (x, y, z)	Docking size (x, y, z)
C3	-9.2	1947	-30, 15, -10	25, 25, 25
C2	-8.9	2174	-44, 19, -31	25, 25, 25
C5	-8.8	457	-50, 32, -6	25, 25, 25
C4	-8.1	941	-10, -3, -9	25, 25, 25
C1	-7.5	3267	-14, 14, -29	25, 25, 25

Ganoderenic Acid B

CurPocket ID	Vina score (kcal/mol)	Cavity volume (Å3)	Center (x, y, z)	Docking size (x, y, z)
C1	-8.6	3267	-14, 14, -29	23, 23, 23
C5	-8.6	457	-50, 32, -6	23, 23, 23
C2	-8.4	2174	-44, 19, -31	29, 23, 23
C4	-8.4	941	-10, -3, -9	23, 23, 23
C3	-2.7	1947	-30, 15, -10	23, 23, 23

Target: Keap1 Kelch Domain / Nrf2 Complex (PDB: 4IN4)

Ganoderic Acid A

CurPocket ID	Vina score (kcal/mol)	Cavity volume (Å3)	Center (x, y, z)	Docking size (x, y, z)
1	-10.4	791	30, -23, 52	25, 25, 25
2	-9.9	824	12, 6, 24	25, 25, 25
3	-9.8	945	22, 22, 46	25, 25, 25
4	-9.3	11411	15, 8, 43	35, 35, 31
5	-8.8	1356	20, 17, 27	25, 25, 25

Ganoderenic Acid B

CurPocket ID	Vina score (kcal/mol)	Cavity volume (Å3)	Center (x, y, z)	Docking size (x, y, z)
1	-10.1	791	30, -23, 52	25, 25, 25
2	-9.6	945	22, 22, 46	25, 25, 25
3	-9.3	11411	15, 8, 43	35, 35, 31
4	-9.2	824	12, 6, 24	25, 25, 25
5	-8.3	1356	20, 17, 27	25, 25, 25

Note. All docking calculations were performed using the CB-Dock2 server (Liu et al., 2022, Nucleic Acids Research, 50, W159-W164, doi:10.1093/nar/gkac394), which employs the CurPocket algorithm for automated binding cavity detection followed by AutoDock Vina-based scoring. For each protein target, the top five predicted cavities are reported in descending order of binding affinity. The cavity exhibiting the most favorable Vina score for each target was selected for the summary comparison presented in Table 2 of the main text, and its location was cross-checked against the reported catalytic or ligand-binding residues of the corresponding protein to confirm biological relevance, as described in Section 2.11 (Molecular Docking Analysis) of the main manuscript.

Table S2. Full ProTox-3.0 toxicological profile of Ganoderic Acid A and Ganoderenic Acid B.

Parameter	Ganoderic Acid A	Ganoderenic Acid B
Predicted oral LD50	9000 mg/kg	9000 mg/kg
Predicted toxicity class	6	6
Hepatotoxicity	Inactive (0.76)	Inactive (0.76)
Neurotoxicity	Inactive (0.93)	Inactive (0.93)
Nephrotoxicity	Active (0.50)	Active (0.50)
Respiratory toxicity	Active (0.96)	Active (0.96)
Cardiotoxicity	Active (0.80)	Active (0.80)
Carcinogenicity	Active (0.55)	Active (0.55)
Immunotoxicity	Inactive (0.52)	Active (0.83)
Mutagenicity	Inactive (0.88)	Inactive (0.88)
Cytotoxicity	Active (0.53)	Active (0.53)
BBB-barrier (toxicity endpoint)	Active (0.59)	Active (0.59)

Note. Toxicological predictions were generated using ProTox-3.0 (Banerjee et al., 2024, Nucleic Acids Research, 52, W513-W520, doi:10.1093/nar/gkae303).
